# Peripheral immune cell response to stimulation stratifies Parkinson’s disease progression from prodromal to clinical stages

**DOI:** 10.1038/s42003-025-08088-7

**Published:** 2025-05-08

**Authors:** Julian R. Mark, Ann M. Titus, Hannah A. Staley, Stephan Alvarez, Savanna Mahn, Nikolaus R. McFarland, Rebecca L. Wallings, Malú Gámez Tansey

**Affiliations:** 1 Department of Neuroscience, University of Florida, College of Medicine, Gainesville, FL 32610 USA; 2 Center for Translational Research in Neurodegenerative Disease, University of Florida, College of Medicine, Gainesville, FL 32610 USA; 3 Department of Neurology and Norman Fixel Institute for Neurological Diseases, University of Florida Health, Gainesville, FL 32608 USA; 4 Department of Neurology and Stark Neuroscience Research Institute, School of Medicine, Indiana University, Indianapolis, IN 46202 USA; 5 McKnight Brain Institute, University of Florida, Gainesville, FL 32610 USA

**Keywords:** Parkinson's disease, Cytokines, Biomarkers

## Abstract

The motor stage of Parkinson’s disease (PD) can be preceded for years by a prodromal stage characterized by non-motor symptoms like REM sleep behavior disorder (RBD), hyposmia, and constipation. Here, we show that multiple stages of idiopathic PD, including the pre-motor prodromal stage, can be stratified according to the inflammatory responses to stimulation of peripheral blood mononuclear cells ex vivo. IFNγ stimulation of isolated monocytes reveals increased stimulation-dependent secretion of TNF, IL-1β, and IL-8 in prodromal PD relative to moderate stage PD. Additionally, T cells stimulated with CD3/CD28 co-stimulatory beads show diminished proinflammatory cytokine secretion in early-moderate PD relative to prodromal. Receiver operating characteristic curves demonstrate that several cytokines produced by stimulated monocytes show high predictive utility for distinguishing prodromal PD individuals from neurologically healthy controls. Moreover, immune stimulation reveals deficits in CD8^+^ T-cell mitochondrial health in moderate PD, with relative mitochondrial health in CD8^+^ T cells being positively correlated with stimulation-dependent secretion of IL-1β, IL-8, and IL-10 in T cells from prodromal PD subjects. Dysregulated mitochondrial health in immune cells may contribute to peripheral inflammation and PD progression, and ex vivo stimulation-based assays have the potential to reveal novel biomarkers for patient stratification and progression with immune endophenotypes.

## Abstract (web summary)

Ex vivo stimulation of isolated monocytes and T cells from peripheral blood immune cells reveals distinct signatures of immune activation across different stages of Parkinson’s disease, including the prodromal stage.

## Introduction

Parkinson’s disease (PD) is a multi-system neurodegenerative disease for which there are no effective disease-modifying therapies. Neuroprotective strategies have been largely ineffective because the majority of dopaminergic neurons in the *substantia nigra pars compacta* (SNpc) have already been lost by the time motor symptoms present and clinical diagnosis can be made^1^. This underscores the need for accessible biomarkers that facilitate early diagnosis and identify patient endophenotypes for superior recruitment and assignment into clinical trials. Significant attention has been directed towards patients with prodromal symptoms of PD, including isolated/idiopathic REM sleep behavior disorder (iRBD) patients, as this disorder is a strong predictive marker of pre-motor prodromal PD^2,3^. RBD is characterized by loss of muscle atonia during REM sleep and the physical acting out of dreams that are often intense or violent^4^. Approximately 80% of iRBD patients will develop a neurodegenerative disease within 10.5 years of iRBD diagnosis, and the plurality (43%) of those who convert will develop PD^2^. Thus, studying individuals with iRBD could reveal novel biomarkers for earlier diagnosis of PD and grant insight into the mechanisms which drive disease progression prior to the onset of classical PD-associated motor symptoms.

Dysregulation in the immune system has long been implicated in the pathogenesis of PD^5^, and this has led to the emergence of peripheral immune dysfunction as a promising mechanism with potentially disease-relevant biomarkers. For example, increased tumor necrosis factor (TNF) receptor expression as well as enhanced production of interferon-gamma (IFNγ) and TNF from T cells have been reported in PD patients^6,7^. Furthermore, circulating monocytes from PD patients display upregulation of genes involved in immune activation, including *HLA-DQB1*, *MYD88*, *REL*, and *TNF*^8^. Together, these findings point towards widespread changes across both the innate and adaptive peripheral immune system in PD. Indeed, it has been shown that the neutrophil-to-lymphocyte ratio (NLR), which serves as a biomarker for systemic inflammation, is significantly correlated with lower levels of dopamine transporter in the striatum of PD patients^9^. Thus, peripheral immune dysregulation may be critical to PD progression, and targeting these mechanisms may improve our ability to deliver personalized treatment plans.

Recent meta-analyses have reported increased blood levels of inflammatory cytokines, such as TNF, IL-1β, and IL-6, in idiopathic PD (iPD) patients compared to controls^10,11^. It has also been found that carriers of PD-associated mutations in *LRRK2* and *GBA1* exhibit increased serum cytokine levels^12,13^, highlighting peripheral immune dysfunction as a common theme shared by both idiopathic and genetic forms of PD. However, circulating cytokine levels are subject to significant variability influenced by circadian rhythm, diet, and environmental exposures^14–17^, which has limited their effectiveness as biomarkers and contributed to heterogeneous reports^18,19^. Moreover, investigations of plasma cytokine levels in prodromal PD have been inconclusive. One study described increased serum TNF levels in iRBD patients^20^, while another reported no differences relative to controls^21^. Therefore, the extent of detectable immune dysfunction in prodromal PD remains to be determined. To facilitate the development of accurate and predictive biomarkers, new approaches are required to overcome background noise with sufficient sensitivity to reveal facets of immune dysfunction that may be difficult to parse apart at baseline.

One approach that our group has demonstrated to help overcome these challenges is to examine differences in immune cell “traits” using stimulus-evoked responses of peripheral immune cells ex vivo^22^. These traits are defined by stimulus-evoked activation and resolution responses, and they can be reproducibly elicited in a controlled ex vivo experiment regardless of exogenous factors. In contrast, the previous literature on baseline cytokine levels describes immune “states”, reflecting only the content of blood cytokines at a single timepoint when the sample is drawn and which can be highly variable^14–17,23,24^. Epidemiologic studies have linked exposure to environmental pathogens with increased long-term risk for developing PD^25^, and this has raised the possibility that an aberrant immune response to stimulation may be more relevant for predicting PD risk than baseline levels of inflammatory factors in the blood. Stimulation-based assays have the potential to provide greater sensitivity, with α-synuclein peptide exposure shown to elicit increased TNF secretion in lymphocytes from PD patients but not from neurologically healthy controls (NHCs)^26^. Immune stimulation also has the advantage of increasing energetic demand^27^, which can highlight deficits in immune cell bioenergetics that have been strongly implicated in PD pathogenesis^28–30^. For example, peripheral blood mononuclear cells (PBMCs) from PD patients show significantly altered mitochondrial respiratory capacity and mitochondrial membrane potential relative to controls^31,32^, as well as downregulation of a number of lysosome/autophagy-related genes including *ULK3*, *ATG2A*, and *HDAC6*^33^. Furthermore, PD monocytes have reduced mitochondrial content relative to controls^34^, and monocyte activity of the lysosomal enzyme glucocerebrosidase is inversely correlated with the severity of motor symptoms after diagnosis^35^. Mitochondrial and lysosomal deficits have yet to be reported in PBMCs at the prodromal stage of PD, but it remains possible that metabolic organelle dysfunction is present before motor symptoms manifest, yet is too subtle to observe with baseline measurements. It is therefore vital to explore how the stage of PD progression may alter immune cell mitochondrial and lysosomal function in response to activation, as this will not only bolster our understanding of PD etiology but potentially provide novel means of identifying at-risk individuals to recruit into suitable trials and for monitoring disease progression.

To close these important gaps in knowledge, we sought to investigate whether the peripheral immune response to stimulation is dysregulated in prodromal patients relative to multiple stages of PD progression. To test this hypothesis, we studied the stimulation-dependent responses of isolated T cells and monocytes from prodromal PD patients, iPD patients at early (within 2 years of diagnosis) and moderate (2-10 years after diagnosis) stage disease, and NHCs. Prodromal PD patients were identified based on referral with endorsement of RBD and the presence of additional prodromal symptoms including hyposmia and constipation (see Methods for inclusion criteria, other clinical information is available in Supplementary Data 1). Isolated monocytes and T cells were stimulated with IFNγ and CD3/CD28 Dynabeads, respectively. The inclusion of early and moderate iPD groups enabled us to capture the dynamic changes in inflammatory responses across the disease spectrum. In addition, cell-type-specific cytokine secretion was evaluated to enhance our ability to detect immune dysfunction traits and determine for the first time if different PBMC subsets display unique patterns of dysregulation in prodromal versus motor PD. Our results show that prodromal PD patients display a distinct signature of immune activation relative to NHCs and clinically diagnosed PD patients, and the immune function of PBMC subsets enables stratification of PD progression across multiple stages of disease.

## Results

### Monocytes from prodromal PD patients display dysregulated stimulation-dependent cytokine secretion

**Fig. 1:**
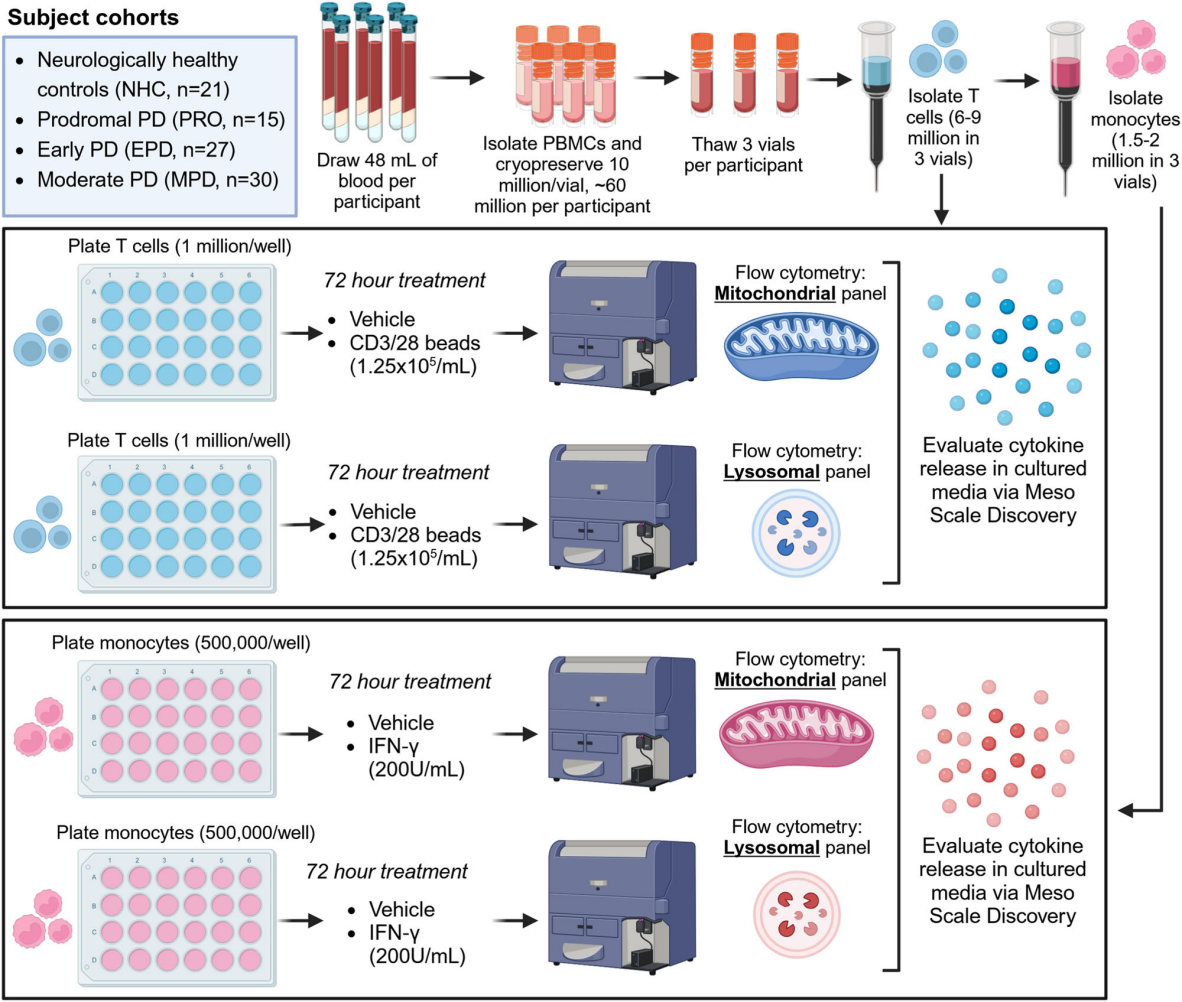
Workflow and experimental design. Whole blood was collected from participants consisting of neurologically healthy controls (NHCs), patients expressing prodromal PD symptoms (REM sleep behavior disorder, hyposmia, preclinical tremor, etc.), early-stage PD patients (diagnosed <2 years prior), and moderate-stage PD patients (diagnosed 2-10 years prior). Peripheral blood mononuclear cells were isolated from whole blood, cryopreserved, and thawed, and subjected to magnetic bead isolations to obtain purified CD3^+^ T cells or purified pan-monocytes. Cells were allowed to rest for 2 h, followed by 72 h incubation in the presence or absence of a stimulation source (monocytes: IFNγ 200 U/mL; T cells: 1.25 × 10^5^ beads/mL of CD3/CD28 Dynabeads). Then cells were assessed via flow cytometry and media was taken for cytokine quantification. The diagram was created with BioRender.com.

To determine if PBMC subsets from iPD patients exhibit differences in stimulation-evoked inflammatory cytokine secretion based on disease progression, we began by collecting PBMCs from patients across the disease spectrum. Whole blood samples were collected from prodromal PD patients (see methods for inclusion criteria) (*n* = 15), early-stage PD patients within 2 years of diagnosis (*n* = 27), moderate-stage PD patients within 2–10 years of diagnosis (*n* = 30), and age- and sex-matched neurologically healthy controls (NHC, *n* = 21). Patients were enrolled at the Norman Fixel Institute for Neurological Diseases at the University of Florida (demographic information of the cohorts is shown in Table 1). From whole blood, PBMCs were isolated and cryopreserved using previously published methods^22^. After PBMCs were thawed, CD3^+^ T cells and pan-monocytes were magnetically isolated, plated, and treated with an immune stimulus (200 U/mL IFNγ for monocytes and 1.25 × 10^5^ beads/mL of CD3/CD28 T-Activator Dynabeads for T cells) or vehicle control for 72 h. The workflow is shown in Fig. 1.

**Table 1:** Demographics for and clinical information for study population

	NHC	PRO	EPD	MPD	*p-*value
N	21	15	27	30	
Age in years, mean (SD)	65.29 (7.90)	68.93 (4.90)	67.19 (6.05)	66.97 (6.27)	0.4161
Sex	10M, 11F	8M, 7F	14M, 13F	16M, 15F	0.9865
Smoking in pack-years, mean (SD)	2.08 (6.01)	7.05 (11.34)	13.82 (21.24)	9.00 (19.73)	0.1298
Caffeine in mg-years, mean (SD)	9678 (6947)	8124 (3869)	8368 (4999)	6636 (4745)	0.2349
NSAID use in mg-years, mean (SD)	634.2 (1924)	529.0 (1073)	937.4 (1598)	1187 (2326)	0.6332
Head injuries, mean (SD)	0.81 (2.6)	0.27 (0.46)	0.48 (0.70)	0.23 (0.50)	0.4411
UPDRS part III motor Score, mean (SD)	N/A	5.25 (10.5)	14.08 (9.27)	21.10 (10.72)	0.0042
Hoehn & Yahr scale, mean (SD)	N/A	N/A	1.74 (0.44)	2.04 (0.52)	0.0494
Disease duration from diagnosis in years, mean (SD)	N/A	N/A	0.65 (0.75)	5.69 (2.63)	<0.0001

Study subjects are matched for age, sex, smoking (pack-yrs), caffeine (mg-yrs), NSAID use (mg-yrs), and head injuries (those with loss of consciousness or requiring medical attention). Chi-square was used for sex. Ordinary one-way ANOVA with Tukey’s adjustmeśnts for multiple comparisons was used for age, smoking, caffeine use, NSAID use, and head injuries. Welch’s *t* test was used for UPDRS part III motor score, Hoehn & Yahr scale, and disease duration.

*NHC* neurologically healthy controls, *PRO* patients with symptoms of prodromal PD, *EPD* early stage PD, *MPD* moderate-stage PD, *NSAID* nonsteroidal anti-inflammatory drug.

**Fig. 2:**
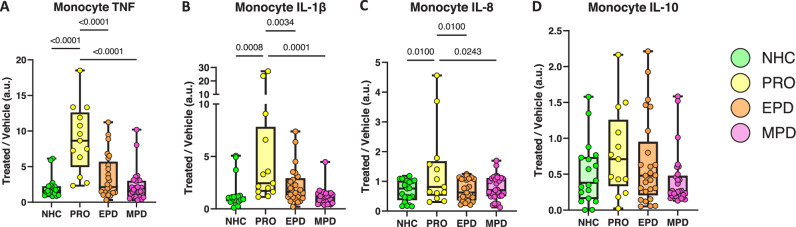
Prodromal PD monocytes exhibit increased stimulation-dependent cytokine secretion. Box plots depicting stimulation-dependent cytokine secretion from isolated monocytes treated with IFNγ. The stimulation-dependent secretion was quantified by normalizing the absolute concentration released in the stimulated condition to the absolute concentration released in vehicle for each participant. Stimulation-dependent secretion of (**A**) TNF, (**B**) IL-1β, (**C**) IL-8, and (**D**) IL-10. Box plots show individual values, median and interquartile range (box), and minimum-maximum range (whiskers). NHC neurologically healthy controls, *n* = 21 biologically independent samples; PRO patients with prodromal PD, *n* = 15 biologically independent samples; EPD patients with early-stage PD, *n* = 27 biologically independent samples; MPD patients with moderate-stage PD, *n* = 30 biologically independent samples. Each symbol represents the measurement from a single individual. The results in (**A**–**D**) were analyzed using one-way ANOVA with Tukey’s corrections for multiple comparisons.

To determine the effects of PD progression on stimulation-evoked innate immune responses, we first assessed the concentrations of inflammatory cytokines in the cultured media from isolated monocytes under baseline and stimulated conditions. Using multiplexed immunoassay platform (Meso Scale Discovery), we observed that IL-8 concentration in the media from vehicle-treated prodromal PD monocytes was significantly reduced relative to other groups (Supplementary Fig. 1A), however absolute concentrations of TNF, IL-1β, and IL-10 were not significantly affected by PD status (Supplementary Fig. 1B–D). Due to significant variability in the data, we proceeded to normalize the concentrations of secreted cytokines in the stimulated condition to the amount secreted in the vehicle condition, allowing each patient to serve as their own normalization factor. We observed that prodromal PD monocytes exhibited significantly increased stimulation-dependent secretion of TNF, IL-1β, and IL-8 relative to all other groups (Fig. 2A–C). No significant differences were observed in IL-10 secretion between patient groups (Fig. 2D). Therefore, anti-inflammatory cytokine secretion was not as significantly modified by PD status as pro-inflammatory pathways. Collectively, such data support a pattern of upregulated stimulation-dependent secretion of proinflammatory cytokines by monocytes in the prodromal stage of PD, which subsequently diminishes in early and moderate stages of PD.

## T cells from moderate stage PD show reduced stimulation-dependent cytokine secretion relative to prodromal PD

**Fig. 3:**
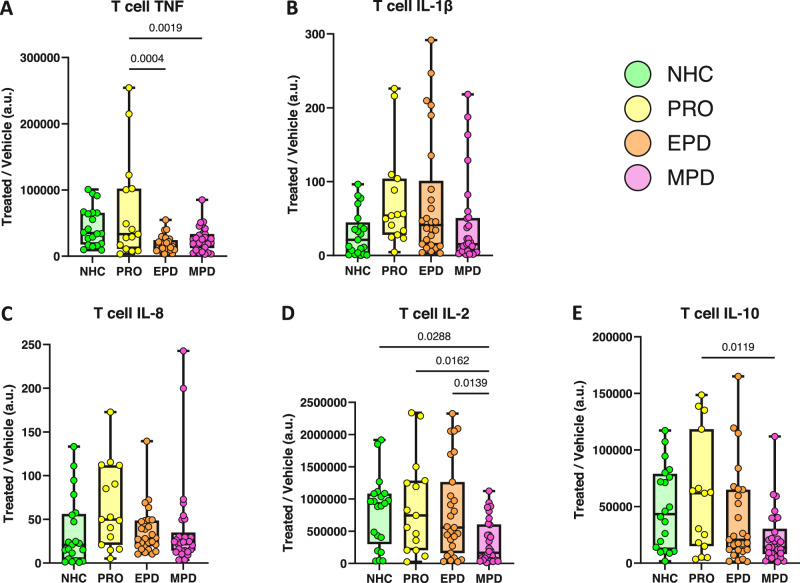
T cell stimulation-dependent cytokine secretion shows diminished response in moderate PD relative to prodromal PD. Box plots depicting stimulation-dependent cytokine secretion from isolated T cells treated with CD3/CD28 Dynabeads. The stimulation-dependent secretion was quantified by normalizing the absolute concentration released in the stimulated condition to the absolute concentration released in vehicle for each participant. Stimulation-dependent secretion of (**A**) TNF, (**B**) IL-1β, (**C**) IL-8, (**D**) IL-2 and (**E**) IL-10. Box plots show individual values, median and interquartile range (box), and minimum-maximum range (whiskers). NHC neurologically healthy controls, *n* = 21 biologically independent samples; PRO patients with prodromal PD, *n* = 15 biologically independent samples; EPD patients with early-stage PD, *n* = 27 biologically independent samples; MPD patients with moderate-stage PD, *n* = 30 biologically independent samples. Each symbol represents the measurement from a single individual. The results in (**A**–**E**) were analyzed using one-way ANOVA with Tukey’s corrections for multiple comparisons.

We next sought to assess if isolated CD3^+^ T lymphocytes displayed similar or distinct patterns of stimulation-dependent cytokine secretion compared to those observed in monocytes. We began by comparing the absolute concentrations of cytokines in the cultured media of T lymphocytes following treatment with vehicle or CD3/CD28 Dynabeads. Absolute levels of TNF secreted from stimulated prodromal PD T cells were significantly increased relative to those from early and moderate PD (Supplementary Fig. 2A). This may indicate that the capacity for T lymphocytes to secrete TNF peaks during prodromal stages and diminishes upon the onset and progression of PD. In addition, we observed that IL-8 secretion from stimulated T cells was highest in early PD and significantly increased relative to NHCs (Supplementary Fig. 2B). Stimulated prodromal PD T cells also showed increased secretion of IL-2 relative to moderate PD and increased secretion of IL-10 relative to NHC, but no differences were observed in IL-1β secretion across patient groups (Supplementary Fig. 2C–E). To mitigate variability, we again normalized the cytokine secretion from the stimulated condition to the vehicle condition. Consistent with the analysis of absolute concentrations, relative stimulation-dependent secretion of TNF was increased from prodromal PD T cells compared to early and moderate PD groups (Fig. 3A). No differences were observed across groups in terms of relative IL-1β and IL-8 secretion (Fig. 3B, C). Interestingly stimulation-dependent secretion of IL-2 from T cells was decreased in moderate PD relative to all other groups (Fig. 3D). In addition, stimulation-dependent IL-10 secretion was reduced in moderate PD relative to prodromal PD (Fig. 3E). Overall, prodromal PD T lymphocytes displayed a pattern of increased stimulation-dependent cytokine secretion compared to moderate PD.

## Stimulation-dependent cytokine secretion has utility for distinguishing patients with prodromal PD from healthy controls

**Fig. 4:**
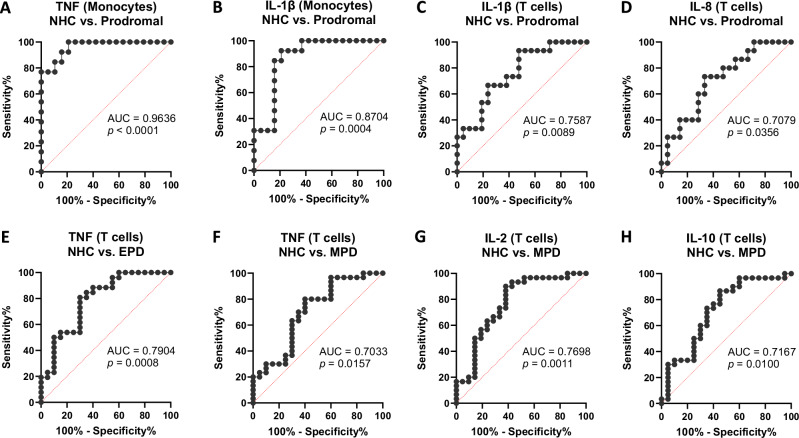
Predictive utility of stimulation-dependent cytokine secretion in healthy controls and patients with different stages of PD. ROC curves for stimulation-dependent cytokine secretion from peripheral immune cells for healthy controls and patients with different stages of PD. Monocyte stimulation-dependent cytokine secretion was calculated by normalizing the concentration secreted after IFN-gamma treatment relative to the vehicle condition. T cell stimulation-dependent cytokine secretion was calculated by normalizing the concentration secreted after CD3/CD28 Dynabead treatment relative to the vehicle condition. **A** TNF secretion from monocytes for NHCs and prodromal PD subjects (AUC = 0.9636, *p* < 0.0001). **B** IL-1β secretion from monocytes for NHCs and prodromal PD subjects (AUC = 0.8704, *p* = 0.0004). **C** IL-1β secretion from T cells for NHCs and prodromal PD subjects (AUC = 0.7587, *p* = 0.0089). **D** IL-8 secretion from T cells for NHCs and prodromal PD subjects (AUC = 0.7079, *p* = 0.0356). **E** TNF secretion from T cells for NHCs and EPD subjects (AUC = 0.7904, *p* = 0.0008). **F** TNF secretion from T cells for NHCs and MPD subjects (AUC = 0.7033, *p* = 0.0157). **G** IL-2 secretion from T cells for NHCs and MPD subjects (AUC = 0.7698, *p* = 0.0011). **H** IL-10 secretion from T cells for NHCs and MPD subjects (AUC = 0.7167, *p* = 0.0100). NHC neurologically healthy controls, *n* = 21 biologically independent samples; Prodromal PD, *n* = 15 biologically independent samples; EPD patients with early-stage PD, *n* = 27 biologically independent samples; MPD patients with moderate-stage PD, *n* = 30 biologically independent samples. All cytokine and patient group comparisons were tested, and only statistically significant ROC curves are shown. Statistical significance for ROC curves defined by AUC > 0.7 and *p* < 0.05.

To determine if peripheral immune cell dysfunction has predictive value for differentiating PD from healthy controls, we conducted receiver-operating characteristic (ROC) analysis of stimulation-dependent cytokine secretion. Monocyte secretion of TNF and IL-1β had predictive value for distinguishing prodromal PD from NHCs (Fig. 4A, B) (TNF: AUC = 0.9636, *p* < 0.0001; IL-1β: AUC = 0.8202, *p* = 0.0031). Prodromal PD could also be distinguished from healthy controls with T cell secretion of IL-1β (AUC = 0.7857, *p* = 0.0089) and IL-8 (AUC = 0.7079, *p* = 0.0356) (Fig. 4C, D). For distinguishing EPD from NHCs, only T cell secretion of TNF showed significant predictive value (AUC = 0.7904, *p* = 0.0008) (Fig. 4E). In addition, T cell secretion of TNF, IL-2, and IL-10 showed predictive value for distinguishing MPD from NHCs (TNF: AUC = 0.7033, *p* = 0.0157; IL-2: AUC = 0.7698, *p* = 0.0011; IL-10: AUC = 0.7167, *p* = 0.0100) (Fig. 4E–G). Collectively, these results suggest that cell-type specific cytokine secretion ex vivo could be a useful component for biomarker-based predictive models aimed at distinguishing healthy individuals from those with PD, including at the prodromal stage.

## Differences in PBMC subtype frequencies reveal changes in immunophenotype based on stage of PD

**Fig. 5:**
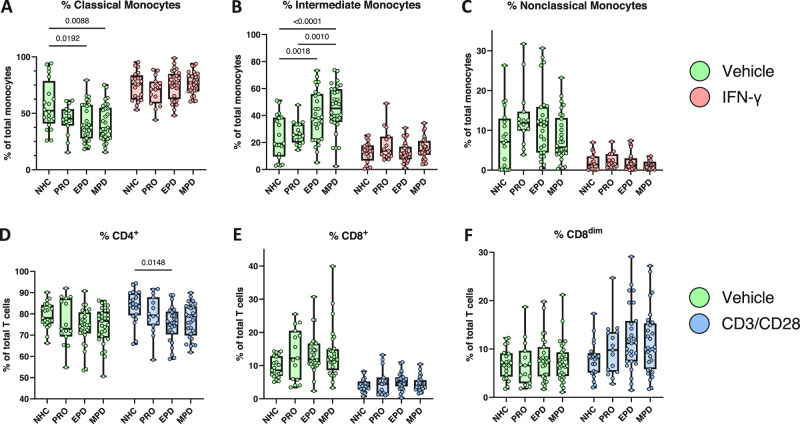
PBMC subtype frequencies across multiple stages of PD. Box plots depicting the frequency of subtypes of monocytes and T cells in PBMCs from NHCs, prodromal PD patients, EPD patients, and MPD patients. **A** Frequency of classical monocytes (CD14^+^CD16^-^) among total monocytes. **B** Frequency of intermediate monocytes (CD14^+^CD16^+^) among total monocytes. **C** Frequency of nonclassical monocytes (CD14^dim^CD16^+^) among total monocytes. **D** Frequency of CD4^+^CD8^-^ among total CD3^+^ T lymphocytes. **E** Frequency of CD4^-^CD8^+^ among total CD3^+^ T lymphocytes. **F** Frequency of CD4^-^CD8^dim^ among total CD3^+^ T lymphocytes. Box plots show individual values, median and interquartile range (box), and minimum-maximum range (whiskers). NHC neurologically healthy controls, *n* = 21 biologically independent samples; PRO patients with prodromal PD, *n* = 15 biologically independent samples; EPD patients with early-stage PD, *n* = 27 biologically independent samples; MPD patients with moderate-stage PD, *n* = 30 biologically independent samples. Each symbol represents the measurement from a single individual. The results in (**A**–**F**) were analyzed using two-way ANOVA with Tukey’s corrections for multiple comparisons. Only within treatment comparisons are shown.

Prior studies have linked PD status to changes in the frequency of PBMC subtypes^9,36^, so we sought to investigate if the observed changes in cytokine secretion were driven by changes in monocyte or T cell sub-population frequencies. To assess this, we stained the isolated monocytes and T cells after treatment for flow cytometry analysis using antibody-fluorophore conjugates for cell-surface markers (see Supplementary Figs. 3, 4 for gating strategy with fluorescence-minus-one controls (FMOCs)). We observed reduced frequencies of classical monocytes (CD14^+^CD16^-^) in vehicle-treated early and moderate PD groups relative to NHCs (Fig. 5A). IFNγ treatment caused an increase in the frequency of classical monocytes in all groups and ablated inter-group differences. Intriguingly, early PD patients displayed significantly elevated proportions of intermediate monocytes (CD14^+^CD16^+^) relative to NHCs in the vehicle condition (Fig. 5B). Furthermore, moderate PD patients showed higher frequencies of intermediate monocytes than both NHC and prodromal groups in vehicle condition. This was surprising because our earlier data suggested that early and moderate PD monocytes as a whole display weaker stimulation-dependent cytokine secretion, yet intermediate monocytes are associated with strong secretion of proinflammatory cytokines^37^. We did not observe differences across patient groups in the frequency of non-classical (CD14^dim^CD16^+^) monocytes (Fig. 5C). IFNγ treatment evoked significant reductions in the frequencies of both intermediate and non-classical monocyte populations (Fig. 5B, C). Raw counts of total monocytes were not significantly different across patient cohorts (Supplementary Fig. 5A). In sum, these results suggest that PD is associated with a shift towards higher frequencies of intermediate monocytes at baseline, away from classical monocytes, but PD status does not interfere with the ability of monocytes to class switch between subtypes following immune stimulation.

Next, we evaluated if the different stages of PD progression were associated with changes in the baseline or stimulation-evoked frequencies of CD4^+^ or CD8^+^ cells among T lymphocytes. CD4^+^ T cell frequencies were similar across groups in the vehicle treated condition, however CD3/CD28 stimulation resulted in a modest but statistically significant decrease in CD4^+^ frequency in early PD relative to NHCs (Fig. 5D). The frequency of CD8^+^ T cells was not significantly different across groups in vehicle or stimulated conditions (Fig. 5E). We also observed a population of CD4^-^ cells which were dimly positive for CD8, distinct from the brighter CD8^+^ population, and we termed this group CD8^dim^ (Supplementary Fig. 3). CD8^dim^ cells have been reported by others^38,39^ and are believed to arise after excessive pathogen burden or immune activation^39^. Bead stimulation had a treatment effect on the frequency of CD8^dim^ T cells (treatment effect, *p* = 0.0001) (Fig. 5F), although there were no significant differences within treatment between patient cohorts.

## Immune activation reveals deficits in CD8^+^ T cell mitochondrial health in moderate PD

**Fig. 6:**
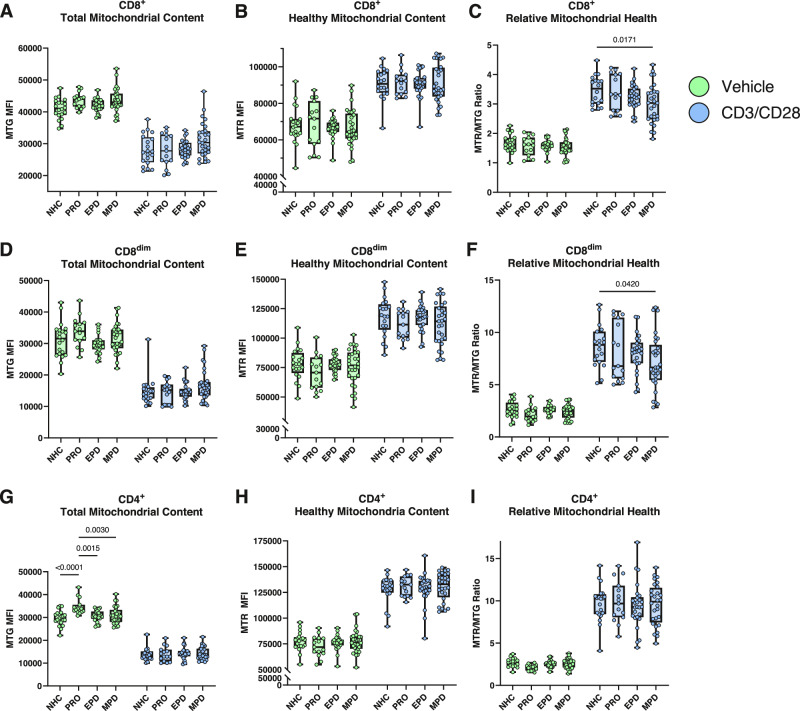
CD8 + T cells from moderate PD patients show impaired relative mitochondrial health following immune stimulation. Box plots depicting the mitochondrial content and mitochondrial health after CD3/CD28 bead stimulation of T cell subsets from NHCs, prodromal PD patients, EPD patients, and MPD patients. **A** Total mitochondrial content of CD8^+^ T lymphocytes. **B** Healthy mitochondrial content with negative membrane potential in CD8^+^ T lymphocytes. **C** Ratio of healthy mitochondrial content (MFI) divided by the total (MFI) in CD8^+^ T lymphocytes. **D** Total mitochondrial content of CD8^dim^ T lymphocytes. **E** Healthy mitochondrial content with negative membrane potential in CD8^dim^ T lymphocytes. **F** Ratio of healthy mitochondrial content (MFI) divided by the total (MFI) in CD8^dim^ T lymphocytes. **G** Total mitochondrial content of CD4^+^ T lymphocytes. **H** Healthy mitochondrial content with negative membrane potential in CD4^+^ T lymphocytes. **I** Ratio of healthy mitochondrial content (MFI) divided by the total (MFI) in CD4^+^ T lymphocytes. Box plots show individual values, median and interquartile range (box), and minimum-maximum range (whiskers). NHC neurologically healthy controls, *n* = 21 biologically independent samples; PRO patients with prodromal PD, *n* = 15 biologically independent samples; EPD patients with early-stage PD, *n* = 27 biologically independent samples; MPD patients with moderate-stage PD, *n* = 30 biologically independent samples. Each symbol represents the measurement from a single individual. The results in (**A**–**I**) were analyzed using two-way ANOVA with Tukey’s corrections for multiple comparisons. Only within treatment comparisons are shown. MTG MitoTracker Green FM, MTR MitoTracker Red CMXRos, MFI median fluorescence intensity.

Growing evidence suggests that cellular metabolic function is intricately connected to immune responses^40^, and mitochondrial deficits have long been implicated in PD pathogenesis^28^. Given that decreased cytokine production in immune cells can be caused by mitochondrial dysfunction^41,42^, we sought to assess if the stage of PD progression was associated with altered mitochondrial health in PBMC subsets. To investigate this, we quantified the median fluorescence intensity (MFI) of MitoTracker probes in stimulated PBMCs using flow cytometry. MitoTracker Green FM (MTG) was used to probe total mitochondrial content^43^, and MitoTracker Red CMXRos (MTR) which stains mitochondria with sufficiently negative membrane potential was used to approximate healthy mitochondrial content^43^. A negative membrane potential is required for ATP production and normal mitochondrial function^44^, thus a reduced MTR MFI indicates a lower quantity of healthy mitochondria. We observed that total mitochondrial content and healthy mitochondrial content were not significantly different across patient groups in CD8^+^ T cells (Fig. 6A, B). To determine if PD progression modulates the relative proportion of healthy mitochondria within the cell, we calculated the MFI ratio of MTR/MTG and compared this ratio across groups. Intriguingly, stimulated CD8^+^ T cells from moderate PD patients showed significantly lower MTR/MTG ratio than NHCs (Fig. 6C). This effect was specific to stimulated cells, as no differences in MTR/MTG ratio were observed at baseline. In the CD8^dim^ population, we again did not observe an effect of PD status on total mitochondrial content or healthy mitochondrial content (Fig. 6D, E). However, CD8^dim^ lymphocytes from moderate PD patients showed significantly reduced relative mitochondrial health after stimulation (Fig. 6F), mirroring the findings from CD8^+^ cells. These results suggest that cytotoxic T cells from moderate PD patients are unable to maintain a high fraction of healthy mitochondria in response to immune activation and increased bioenergetic demand. We then examined CD4^+^ cells and observed that total mitochondrial content in prodromal PD cells was significantly increased relative to all other groups in the vehicle condition (Fig. 6G). No differences were observed across groups in healthy mitochondrial content or relative mitochondrial health in CD4^+^ cells (Fig. 5H, I). These results suggest that prodromal PD may be associated with a greater demand for mitochondria and energy production in CD4^+^ cells at baseline, however, prodromal PD does not disrupt the ability of these cells to regulate mitochondrial health in response to immune activation.

Next, we examined mitochondrial health in monocyte subpopulations, and we observed that PD status was not associated with significant changes in mitochondrial health in classical and intermediate monocytes (Supplementary Fig. 6A–F). In nonclassical monocytes, total mitochondrial content and healthy mitochondrial content also remained unaffected by PD status (Supplementary Fig. 6G, H), but the relative mitochondrial health in moderate PD patients was significantly reduced compared to early PD after stimulation. These data suggest that PD status does not compromise the ability of monocytes to regulate mitochondrial health after stimulation, with the exception of nonclassical monocytes in moderate PD.

## Relative mitochondrial health in CD8^+^ T cells is positively correlated with stimulation-dependent T cell cytokine secretion in prodromal PD

**Fig. 7:**
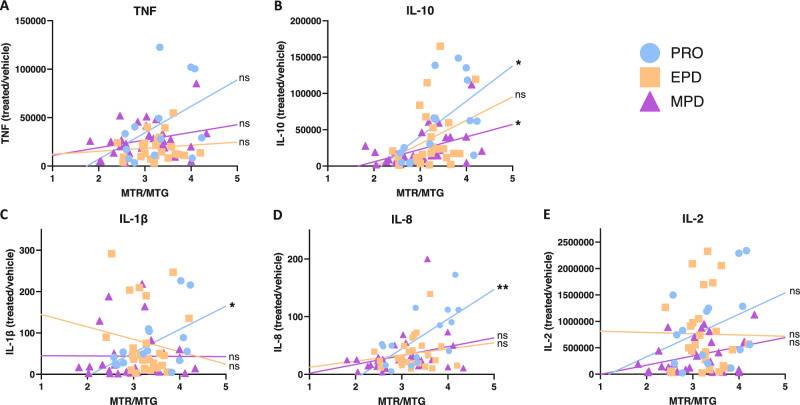
Correlational analysis between relative mitochondrial health in CD8 + T cells and stimulation-dependent cytokine secretion across different stages of PD. The MTR/MTG ratio as a metric of relative mitochondrial health of CD8 + T cells after CD3/CD28 Dynabead stimulation was plotted with linear regression against cytokine secretion of total T cells. **A** TNF secretion [(PRO: *p* = 0.159, *R*^2^ = 0.172), (EPD: *p* = 0.615, *R*^2^ = 0.011), (MPD: *p* = 0.128, *R*^2^ = 0.087)]. **B** IL-10 secretion [(PRO: *p* = 0.025, *R*^2^ = 0.331), (EPD: *p* = 0.118, *R*^2^ = 0.099), (MPD: *p* = 0.010, *R*^2^ = 0.228)]. **C** IL-1β secretion [(PRO: *p* = 0.045, *R*^2^ = 0.275), (EPD: *p* = 0.504, *R*^2^ = 0.020), (MPD: *p* = 0.973, *R*^2^ = 0.000)]. **D** IL-8 secretion [(PRO: *p* = 0.007, *R*^2^ = 0.436), (EPD: *p* = 0.946, *R*^2^ = 0.000), (MPD: *p* = 0.0732, *R*^2^ = 0.118)]. **E** IL-2 secretion [(PRO: *p* = 0.219, *R*^2^ = 0.114), (EPD: *p* = 0.946, *R*^2^ = 0.000), (MPD: *p* = 0.0732, *R*^2^ = 0.118)]. Coefficient and *p*-value based on Pearson correlation. Each symbol represents the measurement from a single individual. PRO patients with prodromal PD, *n* = 15 biologically independent samples; EPD patients with early-stage PD, *n* = 27 biologically independent samples; MPD patients with moderate-stage PD, *n* = 30 biologically independent samples. * signifies that the slope of the line is significantly different from zero (*p < 0.05, **p < 0.01).

Previous reports show that mitochondrial deficits in immune cells can contribute to decreased cytokine secretion^41,45^. Therefore, we sought to explore if the deficits we observed in mitochondrial health following immune stimulation were correlated with deficient cytokine secretion across multiple stages of PD. We had noted that reductions in relative mitochondrial health in moderate PD were specific to cytotoxic T cells, thus we used linear regression to determine the relationship between CD8^+^ MTR/MTG ratio and T cell cytokine secretion. Relative mitochondrial health was not correlated with TNF secretion (Fig. 7A), but it was positively correlated with IL-10 secretion in both prodromal PD and MPD groups (Fig. 7B) (PRO: *p* = 0.025, *R*^2^ = 0.331; MPD: *p* = 0.010, *R*^2^ = 0.228). Relative mitochondrial health was significantly correlated with IL-1β secretion in prodromal PD patients (Fig. 7C) (PRO: *p* = 0.045, *R*^2^ = 0.275). Stimulation-dependent secretion of IL-8 was also correlated with relative mitochondrial health in prodromal PD patients (Fig. 7D) (PRO: *p* = 0.007, *R*^2^ = 0.436), although IL-2 showed no correlation (Fig. 7E). UPDRS part III motor scores in early and moderate PD groups were not associated with T cell cytokine secretion (Supplementary Fig. 7), suggesting that these relationships between mitochondrial health and cytokine secretion are not driven by motor severity. These results indicate that mitochondrial health may represent a useful metric for understanding peripheral immune responses, primarily in prodromal PD patients.

## Monocyte lysosomal function and LRRK2 kinase activity in response to stimulation change with PD progression

**Fig. 8:**
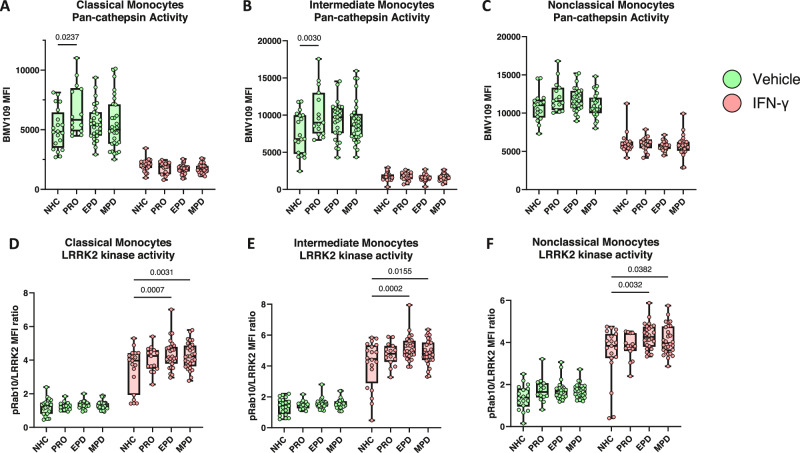
Monocyte subpopulations display distinct profiles of pan-cathepsin and LRRK2 kinase activity based on PD stage. Box plots depicting the pan-cathepsin activity and relative LRRK2 kinase activity after IFNγ stimulation of monocyte subsets from NHCs, prodromal PD patients, EPD patients, and MPD patients. Pan-cathepsin activity was quantified using MFI of BMV109. Relative LRRK2 kinase activity was quantified by dividing the MFI of pRab10 by the MFI of LRRK2. **A** Pan-cathepsin activity of classical monocytes (CD14^+^CD16^-^). **B** Pan-cathepsin activity of intermediate monocytes (CD14^+^CD16^+^). **C** Pan-cathepsin activity of nonclassical monocytes (CD14^dim^CD16^+^). **D** LRRK2 kinase activity of classical monocytes (CD14^+^CD16^-^). **E** LRRK2 kinase activity of intermediate monocytes (CD14^+^CD16^+^). **F** LRRK2 kinase activity of nonclassical monocytes (CD14^dim^CD16^+^). Box plots show individual values, median and interquartile range (box), and minimum-maximum range (whiskers). NHC neurologically healthy controls, *n* = 21 biologically independent samples; PRO patients with prodromal PD, *n* = 15 biologically independent samples; EPD patients with early-stage PD, *n* = 27 biologically independent samples; MPD patients with moderate-stage PD, *n* = 30 biologically independent samples. Each symbol represents the measurement from a single individual. The results in (**A**–**F**) were analyzed using two-way ANOVA with Tukey’s corrections for multiple comparisons. Only within treatment comparisons are shown. MFI median fluorescence intensity.

PD has been linked to deficits in lysosomal degradation in PBMCs^46^, and the severity of motor symptoms in PD patients is inversely correlated with monocyte activity of the lysosomal enzyme glucocerebrosidase^35^. Furthermore, pharmacological inhibition of lysosomal function polarizes immune cells towards a more proinflammatory phenotype and enhances proinflammatory cytokine production^47^. This led us to question whether different stages of PD were associated with stimulation-dependent changes in lysosomal health. To explore this, the MFI of Lysotracker Red DND-99 (LTR), which accumulates in and stains sufficiently acidic lysosomes within cells, was quantified in monocytes and T cells after treatment with our stimulation paradigm. We observed that acidified lysosomal content, quantified from LTR MFI, was not significantly affected by PD status in monocytes, CD4^+^ T cells, or CD8^+^ T cells (Supplementary Fig. 8A–E). However, CD8^dim^ T cells from prodromal PD patients showed increased lysosomal content relative to all other cohorts in vehicle treated condition (Supplementary Fig. 8F). Next, we used the pan-cathepsin fluorescent probe BMV109 to quantify lysosomal cathepsin activity as a marker of lysosomal function in these cells. We first observed that classical monocytes from prodromal PD patients exhibited increased cathepsin activity relative to NHCs in vehicle condition (Fig. 8A). Additionally, intermediate monocytes from prodromal PD patients showed increased cathepsin activity in vehicle condition relative to NHCs (Fig. 8B). No differences in cathepsin activity were observed across groups in nonclassical monocytes (Fig. 8C) or T cell populations (Supplementary Fig. 9). Collectively, these data suggest that PD is associated with an upregulation of baseline lysosomal degradative capacity in specific monocyte subsets, and this upregulation can be detected in the prodromal stage of disease.

Alterations in leucine-rich repeat kinase 2 (*LRRK2*) expression have been heavily implicated in lysosomal biology and function^48,49^, and the gain-of-kinase mutation *LRRK2-G2019S* is one of the most common causes of genetic PD^50^. Additionally, our previous work described changes in the expression of both LRRK2 and phosphorylated Rab10 (pRab10), a LRRK2 substrate indicative of kinase activity, in peripheral immune cells from iPD patients^22^. Recent observations have also defined a role for LRRK2 in suppressing lysosomal degradative activity and regulating cytokine secretion^22,51^. In light of this evidence, we sought to explore the effects of PD progression on LRRK2 expression and kinase activity in stimulated PBMC subsets. Here, IFNγ treatment increased both LRRK2 and pRab10 expression in all monocyte subtypes (Supplementary Fig. 10). Relative LRRK2 kinase activity was assessed by normalizing pRab10 MFI to LRRK2 MFI, and this revealed significantly increased stimulation-dependent LRRK2 kinase activity in all monocyte subtypes from early PD and moderate PD groups relative to NHCs (Fig. 8D–F). Meanwhile, LRRK2 expression and kinase activity in T cell subsets were similar across different stages of PD (Supplementary Fig. 11). Jointly, these experiments indicate that PD is associated with monocyte-specific increases in stimulation-dependent LRRK2 activity.

## Discussion

Emerging literature suggests that immunophenotypic assays have the potential to more clearly define the extent of peripheral immune dysregulation in PD, thereby revealing novel biomarkers and tools for early diagnosis. However, the existence of dysregulated cytokine secretion prior to the onset of motor symptoms in PD remains a matter of contention^20,21^. Furthermore, studies thus far have overlooked the potential for cell-type specific differences in cytokine secretion, leaving open the possibility that specific PBMC subsets exhibit distinct patterns of immune dysfunction in PD. Here we showed that isolated monocytes and T cells from prodromal PD patients display a distinct pattern of immune dysregulation, and that inflammatory responses to stimulation stratify multiple stages of PD progression. Our methodology revealed cell-type specific differences in stimulation-evoked cytokine secretion, PBMC population composition, and facets of mitochondrial and lysosomal health which change dynamically with disease stage.

Our work provides the first evidence to our knowledge that stimulation-evoked cytokine secretion from prodromal PD monocytes is increased relative to controls and individuals with motor symptoms of PD. Previous studies have investigated circulating cytokine levels in iRBD patients and reported heterogenous results^20,21^; however, these studies did not examine the ex vivo response to stimulation nor did they include early or moderate iPD patients for comparison. Our analysis suggests that baseline levels of cytokine secretion are not significantly altered in prodromal monocytes, but instead that these patients display an aberrant upregulation of TNF, IL-1β, and IL-8 secretion in response to immune stimuli. This may represent a potential mechanism whereby increased peripheral inflammation in these individuals contributes to PD conversion and progression. Support for this comes from epidemiological reports that anti-TNF therapy in inflammatory bowel disease patients and ibuprofen use in the general population are associated with a lower incidence of PD^52,53^. Ongoing clinical trials for iRBD and PD include biofluid measurements to assess inflammation^54,55^, however, we propose that stimulation-evoked cytokine secretion may have greater sensitivity and will more accurately report on the cell-type specificity and efficacy of immunomodulatory treatments. Thus, the inclusion of stimulation-dependent immune responses ex vivo will be necessary in future studies to directly investigate peripheral immune dysregulation as a contributing mechanism in PD pathogenesis and progression.

Concurrently, we observed that T lymphocytes from moderate PD patients exhibit reduced secretion of TNF, IL-2, and IL-10 relative to prodromal PD patients. Given that IL-2 controls regulatory T cell maturation and proliferation^56^, our results suggest that regulatory T cell maturation may be disrupted in moderate PD consistent with current literature^57^. TNF and IL-2 are generally considered pro-inflammatory, in contrast to IL-10 which is generally considered anti-inflammatory and typically secreted alongside pro-inflammatory markers to resolve an inflammation response. Therefore, these findings suggest a nuanced pattern of dysregulation in moderate PD. One possible driver of immune dysregulation in MPD is bioenergetic insufficiency secondary to mitochondrial dysfunction. This could limit the ability of T lymphocytes from MPD patients to effectively upregulate cytokine production after exposure to immune stimuli. Indeed, we observed that cytotoxic T cells from MPD patients displayed poorer relative mitochondrial health after stimulation compared to NHCs. Similarly, mitochondrial impairment in neurons is widely reported in idiopathic and genetic forms of PD^58–60^, and reduced mitochondrial respiration has been shown to elicit T cell exhaustion in chronic infections^41^. Interestingly, UPDRS part III motor scores did not correlate with stimulation-dependent cytokine secretion. Current perspectives acknowledge that additional metrics beyond motor progression are necessary to appreciate emerging sub-types of PD^61–63^, and a combination of biochemical markers with clinical measures may be necessary to precisely identify patient endophenotypes for reduced heterogeneity in clinical trials^64,65^.

Underlying differences in immune priming between prodromal PD and MPD immune cells could account for the observed stimulation-dependent responses we observed. Immunogenic mitochondrial damage-associated molecular patterns (DAMPs) and mitochondrial DNA are known to activate TLR4^66,67^, and it is possible that these are present at higher concentrations in peripheral immune cells from prodromal PD patients. In support of this, a recent study found that fibroblasts from iRBD patients who converted to PD had significantly increased mitochondrial fragmentation relative to controls, and iRBD patients who did not convert showed similar but milder alterations^68^. Therefore, we posit that mitochondrial fragmentation predisposes towards an increased immune response early in the disease course, but persistent accumulation of mitochondrial dysfunction eventually overwhelms the adaptive ability of these cells and leads to immune exhaustion later in PD. Future research should seek to evaluate mitochondrial morphology in immune cells across the PD spectrum, as pharmacologic interventions aimed at improving mitochondrial biogenesis and enhancing mitophagy represent novel and exciting avenues that should be explored to delay disease progression.

Flow cytometry analysis revealed that moderate PD patients show a reduced frequency of classical monocytes and an increased frequency of intermediate monocytes compared to NHCs. Our findings are consistent with those from Thome et al. who reported identical changes in monocyte subtype frequencies in PD patients^57^. Intermediate monocytes have been reported to secrete higher levels of proinflammatory cytokines than classical monocytes in some contexts^69,70^, which seems at odds with our findings that showed moderate PD monocytes have diminished stimulation-dependent cytokine secretion relative to prodromal PD. One potential explanation is that the shift towards increased numbers of intermediate monocytes is a compensatory response to underlying functional deficits in these individuals. In addition, we observed that immune stimulation of T lymphocytes led to an increased frequency of CD8^dim^ cells in most groups; however, there was no effect of patient cohort on CD8^dim^ frequency. Consequently, our results do not indicate that PD status or disease progression significantly affects stimulation-dependent downregulation of CD8 downregulation in T lymphocytes. Our flow panels did not differentiate more specialized cell types such as helper, regulatory, or natural killer T cells, which will be important in future efforts to comprehensively describe how PD stage affects peripheral immune populations. Thus, further efforts using scRNAseq or similar methodologies capable of garnering deep cell-type-specific data to explore differences across these PBMC subsets should be considered.

Our study also demonstrated increased pan-cathepsin activity in classical and intermediate monocytes from prodromal PD patients at baseline relative to NHCs. Upregulation of cathepsins has been reported in multiple PD models^71,72^; however, our work provides the first evidence that lysosomal function as measured by pan-cathepsin activity is upregulated in the prodromal stage. Cathepsin activity is known to modulate autophagic flux^73^, therefore this may represent an increased demand for lysosomal degradation and autophagy prior to the onset of motor symptoms. Indeed, autophagic flux increases to compensate for mitochondrial defects^74^, and mitochondrial membrane potential is a known regulator of autophagic flux^75^. Lysosomal and mitochondrial dynamics are extensively linked to one another, so additional research is necessary to determine if immune cell mitochondrial dysfunction in PD is a downstream consequence of disturbances in lysosomal activity. In sum, our findings point to observable changes in lysosomal function in prodromal PD patients, suggesting that peripheral immune cell dysregulation is present prior to motor manifestations of PD. Moreover, these deficits can be ascertained non-invasively to potentially stratify and enroll patients who are more likely to benefit from interventions targeting mitochondrial and lysosomal function.

In addition, we observed significantly increased LRRK2 kinase activity in stimulated monocytes from EPD and MPD patients relative to NHCs. These findings imply a convergence of both idiopathic and genetic forms of PD (e.g. *LRRK2-G2019S* PD) on dysregulated LRRK2 kinase activity in immune cells. Inhibition of LRRK2 kinase activity has been proposed as a potential approach to combat neuroinflammation in iPD^76^, and our group has previously demonstrated that LRRK2-targeting anti-sense oligonucleotide therapy reduces inflammatory cytokine secretion in *Lrrk2-G2019S* peripheral macrophages^77^. Therefore, peripheral immune cell stimulation ex vivo may be useful for noninvasively evaluating patient responsiveness to pharmacologic interventions aimed at modulating LRRK2 kinase activity. Further investigation is necessary with pharmacologic inhibitors, because in the current study we cannot conclusively distinguish whether increased LRRK2 kinase activity is compensating for or contributing to immune dysregulation in the clinical stages of iPD.

Some limitations to this study must be noted. The estimated risk for patients with iRBD to develop PD is 43%, with the next most likely course being a 25% risk for dementia with Lewy bodies^2^. Therefore, iRBD is not entirely specific for prodromal PD, and our findings may represent dysregulation in a common pathway shared by multiple neurodegenerative diseases. In addition, our study design is based on cross-sectional analyses and did not include follow-up sampling for the enrolled prodromal PD patients; thus, we cannot conclusively determine which of these patients will ultimately convert to motor PD. The group sizes used in this study are close to those used in similar stimulation-based assays^78,79^, but it will be vital for future longitudinal studies to be conducted with larger cohorts to replicate these findings and correlate these immune markers with eventual PD conversion rates. Identification of individuals with prodromal PD features remains a challenge for the field and is inherently limited by the capacity of the movement disorders clinic to enroll them into an observational trial. Nonetheless, it will be important for these results to be validated by independent research groups by replication in a different cohort.

Here, we tested a single stimulation duration which was chosen based on preliminary optimization and to conserve the limited number of precious samples available. However, peak cytokine production does not occur at a singular time point^80^, thus it will be valuable for future studies to investigate earlier time points to determine how the kinetics of cytokine secretion may be impacted by the stage of PD progression. It is also possible that the extended stimulation time introduced variability in the forms of cytokine degradation and expansion or depletion of specific immune cell subsets, which limits the conclusions that can be drawn. On the other hand, to examine stimulation-dependent responses on longer time scales such as weeks or months, other preclinical models such as animal models with chronic LPS injection may be more appropriate than acute ex vivo stimulation of human PBMCs. Further effort employing these models may help to determine the interactions between PD progression and the immune system’s ability to respond to long-duration insults. Our findings suggest that T cells from moderate PD patients exhibit deficits in production of cytokines such as IL-2 which is generally consistent with an immune exhaustion phenotype^81^. We were unable to include additional fluorophores for bona-fide immune exhaustion markers (e.g. TIM3, PD-1, CTLA-4) due to limitations in the number of flow cytometry channels available, therefore it will be necessary for future studies to evaluate expression of these markers. Our results suggest that dysregulated mitochondrial health can correlate with immune function, but orthogonal measures of mitochondrial health including oxygen consumption rate and mitochondrial turnover will be important for future studies. Larger scope analyses such as proteomics and metabolomics are warranted for future efforts to define the exact molecular mechanisms driving aberrant immune response in prodromal PD in greater depth.

In summary, this work demonstrates that peripheral blood immune cells from prodromal PD patients display a distinct pattern of stimulation-dependent immune dysregulation relative to NHCs and clinically diagnosed PD groups. These findings hold significant potential to advance both scientific understanding and clinical practice for PD. First, our use of sensitive stimulation-dependent assays and focus on cell-type-specific cytokine secretion has allowed us to detect a specific pattern of peripheral immune dysregulation in prodromal PD patients, thereby addressing inconsistencies in previous studies. Second, our finding that T cell mitochondrial health correlates with stimulation-dependent cytokine secretion in prodromal PD patients reveals a novel target for therapeutic intervention. Early intervention to rescue mitochondrial deficits may help mitigate excessive inflammation prior to the development of motor PD, while in advanced stages it may be a crucial target to combat immune exhaustion or senescence. Lastly, we recommend the expanded use of ex vivo stimulation-based peripheral immune cell biomarkers, such as stimulation-dependent cytokine secretion and measures of mitochondrial health, to distinguish unique immunophenotypes for PD patients. It is becoming increasingly apparent that disease duration and severity of motor symptoms are not sufficient to describe the spectrum of PD progression, and the inability of the field to capture this heterogeneity across patients has been a barrier to success for clinical trials. Our findings may help direct future research towards the convergence of immune function and bioenergetics as the field continues to search for clinically relevant PD biomarkers.

## Materials and methods

### Human subjects

Ethical compliance: this study was reviewed and approved by the University of Florida Institutional Review Board (IRB202002639). Participants provided written informed consent to participate. All ethical regulations relevant to human research participants were followed. Blood was initially collected from healthy volunteers to establish and optimize assay parameters. The prodromal PD cohort (*n* = 15) was recruited on the basis of referral with patient endorsement of RBD diagnosis, and the majority of patients expressed some additional prodromal symptoms including hyposmia and constipation. The frequencies of these additional symptoms and other clinical information are available in Supplementary Data 1. Early PD (*n* = 27), moderate PD (*n* = 30), and age-matched neurologically healthy controls (*n* = 21) were also recruited through the Norman Fixel Institute for Neurological Diseases at the University of Florida for this study. Early PD patients were <2 years post-diagnosis with <1 year on PD medications. Moderate PD patients were between 2 and 10 years post-diagnosis. Subjects were excluded based on age (younger than 30 and over 80 years of age), known familial PD mutations and/or other known neurological, chronic or recent infections, or autoimmune comorbidities. Subjects were genotyped for the *LRRK2-G2019S* mutation (Life Technologies #4351378, Grand Island, NY) and excluded from this study if they were shown to be mutation carriers.

During recruitment, a family history and environmental questionnaire was used to assess the history of disease and inflammation/immune-relevant environmental exposures and comorbidities. Caffeine use, non-steroidal anti-inflammatory drug (NSAID) use, and nicotine exposure were calculated as mg-years, mg-years, and pack-years, respectively. The study populations were balanced with respect to risk factors for PD, including age, smoking, non-steroidal anti-inflammatory drug use, and caffeine intake (Table 1).

### Peripheral blood mononuclear cell (PBMC) isolation and cryopreservation

Cell isolation was accomplished using BD Vacutainer CPT Cell Preparation Tube with Sodium Citrate (BD Biosciences, 362761) using previously established methods. Approximately 6 CPT tubes, each containing 8 mL of blood, were collected from each participant. CPT tubes were inverted 8–10 times and centrifuged at room temperature at 1500 × *g* for 20 min at room temperature. The PBMC-enriched layer was transferred to a new 50 mL conical tube and MACS buffer (PBS, 0.5% bovine serum albumin, 20 mM EDTA, pH 7.2) was added to a final volume of 50 mL, followed by centrifugation at 1800 × *g* for 10 min at room temperature. Following removal of the supernatant, PBMCs were resuspended in 10 mL MACS buffer and counted on a hemocytometer using Trypan blue (1:20 dilution) exclusion to ascertain viability.

Next, to cryopreserve the samples, PBMCs were centrifuged for 5 min 1800 × *g* at room temperature. Supernatant was aspirated and cell pellets were gently resuspended in cryopreservation media (54% RPMI 1640, 36% FBS, 10% DMSO) at a final concentration of 1 × 10^7^ cells/mL in cryovials (Simport, T311-2). Cryovials were placed in a room-temperature Mr. Frosty freezing container with isopropanol as per the manufacturer’s instructions and stored at −80 °C overnight. After overnight storage at −80 °C, the next day cryovials were removed from freezing containers and placed into liquid nitrogen for long-term storage.

### Cryorecovery of isolated PBMCs

For cryorecovery, cryovials of PBMCs were retrieved from liquid nitrogen, rapidly thawed in a water bath at 37 °C, and rapidly added to 25 mL of 37 °C filter sterilized complete culture media (RPMI 1640 media, 10% low endotoxin heat-inactivated FBS, 1 mM Penicillin-Streptomycin). PBMCs were pelleted via centrifugation at 300 × *g* for 10 min at room temperature. Pellets were gently resuspended in 10 mL of 37 °C MACS buffer (PBS, 0.5% bovine serum albumin, 20 mM EDTA, pH 7.2), then viability and cell count were obtained with a hemocytometer using Trypan blue (1:20 dilution) exclusion to ascertain viability.

### Ex vivo isolation of CD3 + T cells and pan monocytes from PBMCs

Following cryorecovery, CD3^+^ T cells were isolated from total PBMCs using REAlease® CD3 MicroBead Kit, human (Miltenyi, 130-117-038) following the manufacturer’s instructions with slight modifications. PBMCs were centrifuged at 300 × *g* for 10 min at room temperature, supernatant was aspirated, and pellets were gently resuspended in 40 μL of separation buffer (PBS, 0.5% bovine serum albumin, 2 mM EDTA, pH 7.2) per 1 × 10^7^ total cells. 10 μL of REAlease CD3-Biotin were added per 1 × 10^7^ total cells, mixed well, and samples were incubated at room temperature for 5 min. 100 μL of REAlease Anti-Biotin Microbeads (CD3, human) were added per 1 × 10^7^ total cells, mixed well, and samples were incubated at room temperature for 5 min. Samples were diluted to a total volume of 2 mL with separation buffer then passed through pre-wetted LS columns (Miltenyi, 130-042-401) in a QuadroMACS™ Separator (Miltenyi, 130-091-051). Columns were washed 3 times with 3 mL of separation buffer, and the flow-through was set aside at 4 °C for isolation of monocytes. LS columns were removed from the magnetic separator and flushed twice with 5 mL of REAlease Bead Release buffer to release bead-bound CD3^+^ cells. CD3^+^ samples were mixed well and incubated at room temperature for 5 min. Then, CD3^+^ samples were centrifuged at 300 × *g* for 10 min at 4 °C, supernatant was aspirated, pellets were gently resuspended in 5 mL separation buffer. 100 μL of REAlease Release Reagent was added to each sample, mixed well, and then CD3^+^ cells were counted using a hemocytometer with Trypan blue (1:20 dilution) to ascertain viability.

Monocytes were isolated from the flow-through of CD3^-^ cells using Pan Monocyte Isolation Kit, human (Miltenyi, 130-096-537) following the manufacturer’s instructions with slight modifications. Cells were centrifuged at 300 × *g* for 10 min at 4 °C, supernatant was aspirated, and pellets were gently resuspended in 45 μL of cold separation buffer per 1 × 10^7^ total cells. 15 μL of FcR blocking reagent and 18.75 μL of Biotin-antibody cocktail was added per 1 × 10^7^ total cells, samples were mixed well, and then cells were incubated for 5 min at 4 °C. 45 μL of cold separation buffer and 30 μL of Anti-Biotin Microbeads were added per 1 × 10^7^ total cells, samples were mixed well, and then cells were incubated for 5 min at 4 °C. Samples were diluted to a total volume of 2 mL with cold separation buffer then passed through pre-wetted LS columns in a QuadroMACS™ Separator. Columns were washed 3 times with 3 mL of separation buffer, and the flow-through containing purified monocytes was counted on a hemocytometer using Trypan blue (1:20 dilution) to ascertain viability.

### Ex vivo T cell and monocyte cell culture plating and treatments

T cells were diluted to a final concentration of 1 × 10^6^ per mL in 1 mL complete culture media in 24-well plates and allowed to rest for 2 h at 37 °C, 5% CO_2_, 95% relative humidity. After resting, cells were treated with either vehicle or 1.25 × 10^5^ beads/mL Dynabeads™ Human T-Activator CD3/CD28 (Gibco, 11161D) for 72 h at 37 °C, 5% CO_2_, 95% relative humidity.

Monocytes were diluted to a final concentration of 5 × 10^5^ per mL in 1 mL complete culture media in 24-well plates and allowed to rest for 2 h at 37 °C, 5% CO2, 95% relative humidity. After resting, cells were treated with either vehicle or 200U human IFNγ (Peprotech, 300-02) for 72 h at 37 °C, 5% CO_2_, 95% relative humidity.

### Live cell flow cytometry assay for mitochondrial health and pan-cathepsin activity

After the 72-h stimulation, cells were harvested and centrifuged at 300 × *g* for 10 min at 4 °C. Supernatant was collected to quantify cytokine secretion (described later). Cell pellets were gently resuspended in 400 μL of cold PBS, then 200 μL per sample were transferred to 2 separate v-bottom 96-well plates (Sigma, CLS3896-48EA), one for the live cell panel and another for the fixed panel. Both panels were run on the same day. Samples in the live cell panel were centrifuged at 300 × *g* for 5 min at 4 °C. Cells were resuspended in 200 μL of complete growth media containing 1 μM MitoTracker™ Red CMXRos (Invitrogen, M7512),1 μM MitoTracker™ Green FM (Invitrogen, M7514), and 1 μM BMV109 Pan Cathepsin Probe (Vergent Biosciences, 40200-200). Cells were incubated for 1 h at 37 °C in the dark. Samples were centrifuged at 300 × *g* for 5 min at 4 °C. Cell pellets were resuspended in PBS and washed × 2 by centrifugation at 300 × *g* for 5 min at 4 °C. Cells were resuspended in 50 μL of Live/Dead Fixable Violet stain (diluted 1:2000 in PBS, Invitrogen, L34962) and incubated in the dark at room temperature for 30 min. Cells were centrifuged at 300 × *g* for 5 min at 4 °C washed in PBS × 2. Cells were resuspended in 50 μL of PBS containing diluted antibodies (see Supplementary Table 1 for T cell panel, see Supplementary Table 2 for monocyte panel) and incubated in the dark at 4 °C for 20 min. Cells were centrifuged at 300 × *g* for 5 min at 4 °C washed in FACS buffer (PBS, 0.5 mM EDTA, 0.1% sodium azide) × 3. Cells were analyzed via flow cytometry on a FACSymphony™ A3 cytometer (BD Biosciences). Data were analyzed using FlowJo version 10.10.0 software (BD Biosciences). When validating all flow cytometry panels and antibodies, fluorescence minus one controls (FMOCs) were used to set gates and isotype controls were used to ensure antibody-specific binding.

### Fixed cell flow cytometry and staining for lysosomal health and LRRK2 activity

Samples in the fixed cell panel were centrifuged at 300 × *g* for 5 min at 4 °C. Cells were resuspended in 200 μL of complete growth media, and LysoTracker™ Red DND-99 (Invitrogen, L7528) was added to reach a final concentration of 500 nM for T cells or 200 nM for monocytes. Cells were incubated for 1 h at 37 °C in the dark. Samples were centrifuged at 300 × *g* for 5 min at 4 °C. Cell pellets were resuspended in PBS and washed × 2 by centrifugation at 300 × *g* for 5 min at 4 °C. Cells were resuspended in 50 μL of Live/Dead Fixable Violet stain (diluted 1:2000 in PBS, Invitrogen, L34962) and incubated in the dark at room temperature for 30 min. Cells were centrifuged at 300 × *g* for 5 min at 4 °C washed in PBS × 2. Cells were resuspended in 50 μL of PBS containing diluted antibodies (see Supplementary Table 1 for T cell panel, see Supplementary Table 2 for monocyte panel) and incubated in the dark at 4 °C for 20 min. Cells were centrifuged at 300 × *g* for 5 min at 4 °C and washed × 2 in PBS. Cells were re-suspended and fixed in 100 μL of 1% paraformaldehyde (PFA) at 4 °C in the dark for 30 min. Cells were washed 2 × with PBS, then resuspended in 100 μL of permeabilization buffer (eBiosciences, 00-8333-56) and incubated on ice for 15 min. Anti-pT73 Rab10 antibody (Abcam, ab241060) was added to each well at 0.55 μg per well and incubated at room temperature and protected from light for 30 min. Cells were centrifuged at 300 × *g* for 5 min at 4 °C washed in PBS × 2. Cells were resuspended in 100 μL of PBS containing 1% normal goat/donkey serum, 2% BSA and 1:1000 AF488 donkey anti-rabbit secondary (Thermo Fisher, A-21206) and incubated at room temperature and protected from light for 30 min. Cells were centrifuged at 300 × *g* for 5 min at 4 °C washed in PBS × 2. Cells were resuspended in 100 μL of PBS containing 1% normal goat/donkey serum, 2% BSA 1:100 anti-LRRK2 AF700 antibody and incubated at 4 °C covered for 20 min. Cells were centrifuged at 300 × *g* for 5 min at 4 °C, and then washed in FACS buffer × 3. Cells were analyzed via flow cytometry on a FACSymphony™ A3 cytometer (BD Biosciences). Data were analyzed using FlowJo version 10.10.0 software.

### Cytokine quantification

V-PLEX custom Human Biomarkers kit (Meso Scale Discovery (MSD), K151ARH-2) was used to quantify TNF, IL-1β, IL-2, IL-8, and IL-10 concentrations in conditioned media from cultured T cells and monocytes. Media was diluted 1:4 with MSD kit diluent and incubated in duplicate at room temperature in the provided MSD plate with capture antibodies for 2 h as per the manufacturer’s instructions. Plates were then washed × 3 with PBS with 0.05% Tween-20 and detection antibodies conjugated with electrochemiluminescent labels were added and incubated at room temperature for another 2 h. After 3 × washes with PBS containing 0.05% Tween-20, MSD buffer was added and the plates were loaded into the QuickPlex MSD instrument for quantification.

### Statistics and reproducibility

Data and statistical analyses were performed using GraphPad Prism 10. For assessing differences between groups, data were analyzed by either one-way or two-way analysis of variance (ANOVA), or by *t*-test. In instances when data did not fit parametric assumptions, Kruskal–Wallis non-parametric ANOVA was used. *Post-hoc* tests following ANOVAs were conducted using Tukey’s method for correcting for multiple comparisons. For assessing relationships between read-outs, data were analyzed by Pearson’s *r*. In instances when data did not fit parametric assumptions, Spearman’s rank was used to assess relationships between variables. Two-tailed levels of significance were used and *p* < 0.05 was considered statistically significant. Graphs are depicted by means ± standard error of the mean (SEM).

### Reporting summary

Further information on research design is available in the Nature Portfolio Reporting Summary linked to this article.

## Supplementary information

Supplementary Information

Description of Additional Supplementary Files

Supplementary Data 1

Supplementary Data 2

Reporting Summary

## Data Availability

Source data are provided with this paper in Supplementary Data 2. Other data are available in Supplementary Information and Supplementary Data 1.
