# Supplementary material for: Peripheral immune cell response to stimulation stratifies Parkinson’s disease progression from prodromal to clinical stages
Source: Commun Biol. 2025 May 8;8:716. doi: 10.1038/s42003-025-08088-7 (PMC12062209; doi:10.1038/s42003-025-08088-7)
Supplement: Supplementary file 1 — Supplementary Information [file 42003_2025_8088_MOESM1_ESM.pdf]

Supplementary Fig. 1: Monocyte absolute cytokine secretion

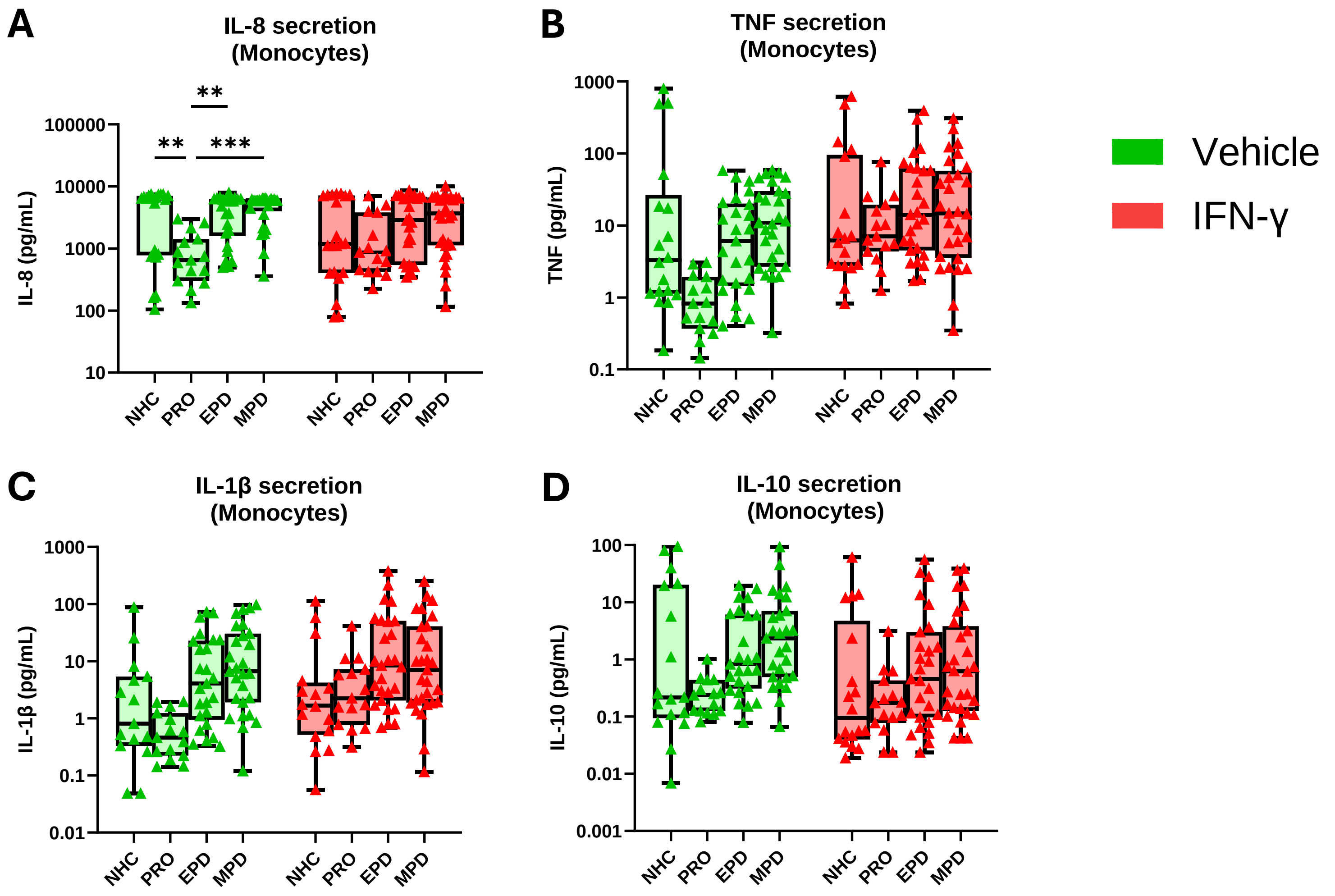

Supplementary Fig. 1: Monocyte absolute cytokine secretion. Box plots depicting absolute concentrations of secreted inflammatory cytokines from isolated monocytes treated with IFNγ. Monocytes were obtained from NHCs, prodromal PD patients, EPD patients, and MPD patients. Absolute concentrations of secreted (**A**) IL-8, (**B**) TNF, (**C**) IL-1β, and (**D**) IL-10. Box plots show individual values, median and interquartile range (box), and minimum-maximum range (whiskers). NHC neurologically healthy controls, n = 21 biologically independent samples; PRO patients with prodromal PD, n = 15 biologically independent samples; EPD patients with early-stage PD, n = 27 biologically independent samples; MPD patients with moderate-stage PD, n = 30 biologically independent samples. Each symbol represents the measurement from a single individual. The results in **A-D** were analyzed using two-way ANOVA with Tukey's corrections for multiple comparisons. Only within treatment comparisons are shown. Statistical significance is denoted by asterisks (* $p < 0.05$, ** $p < 0.01$, *** $p < 0.001$).

Supplementary Fig. 2: T cell absolute cytokine secretion

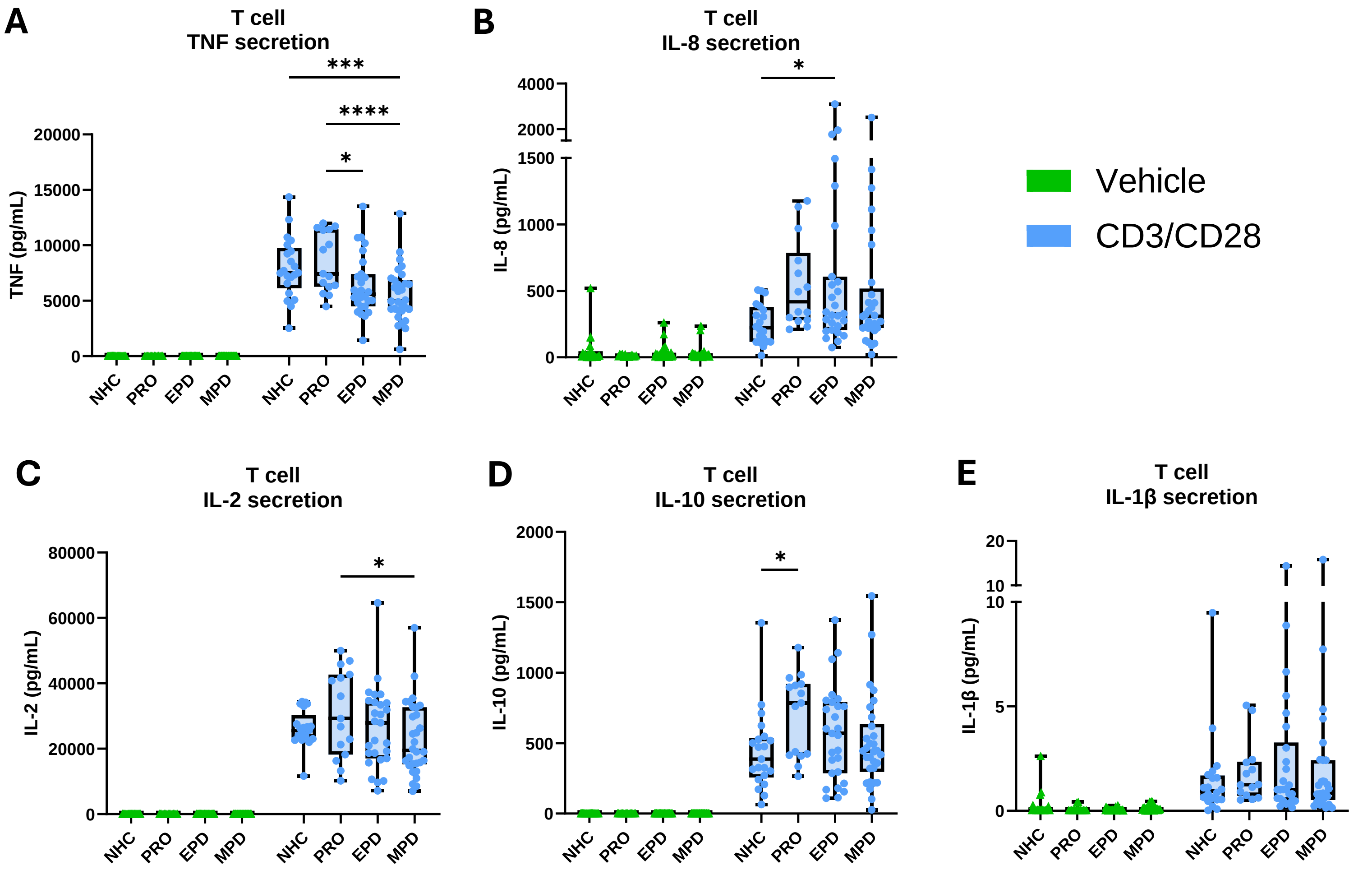

Supplementary Fig. 2: T cell absolute cytokine secretion. Box plots depicting absolute concentrations of secreted inflammatory cytokines from isolated T cells treated with CD3/CD28 Dynabeads. T cells were obtained from NHCs, prodromal PD patients, EPD patients, and MPD patients. Absolute concentrations of secreted (**A**) TNF, (**B**) IL-8, (**C**) IL-2, (**D**) IL-10, and (**E**) IL-1β. Box plots show individual values, median and interquartile range (box), and minimum-maximum range (whiskers). NHC neurologically healthy controls, *n* = 21 biologically independent samples; PRO patients with prodromal PD, *n* = 15 biologically independent samples; EPD patients with early-stage PD, *n* = 27 biologically independent samples; MPD patients with moderate-stage PD, *n* = 30 biologically independent samples. Each symbol represents the measurement from a single individual. The results in **A-E** were analyzed using two-way ANOVA with Tukey's corrections for multiple comparisons. Only within treatment comparisons are shown. Statistical significance is denoted by asterisks (* $p < 0.05$, ** $p < 0.01$, *** $p < 0.001$).

Supplementary Fig. 3: Flow cytometry gating strategy for T cells using fluorescence-minus-one controls

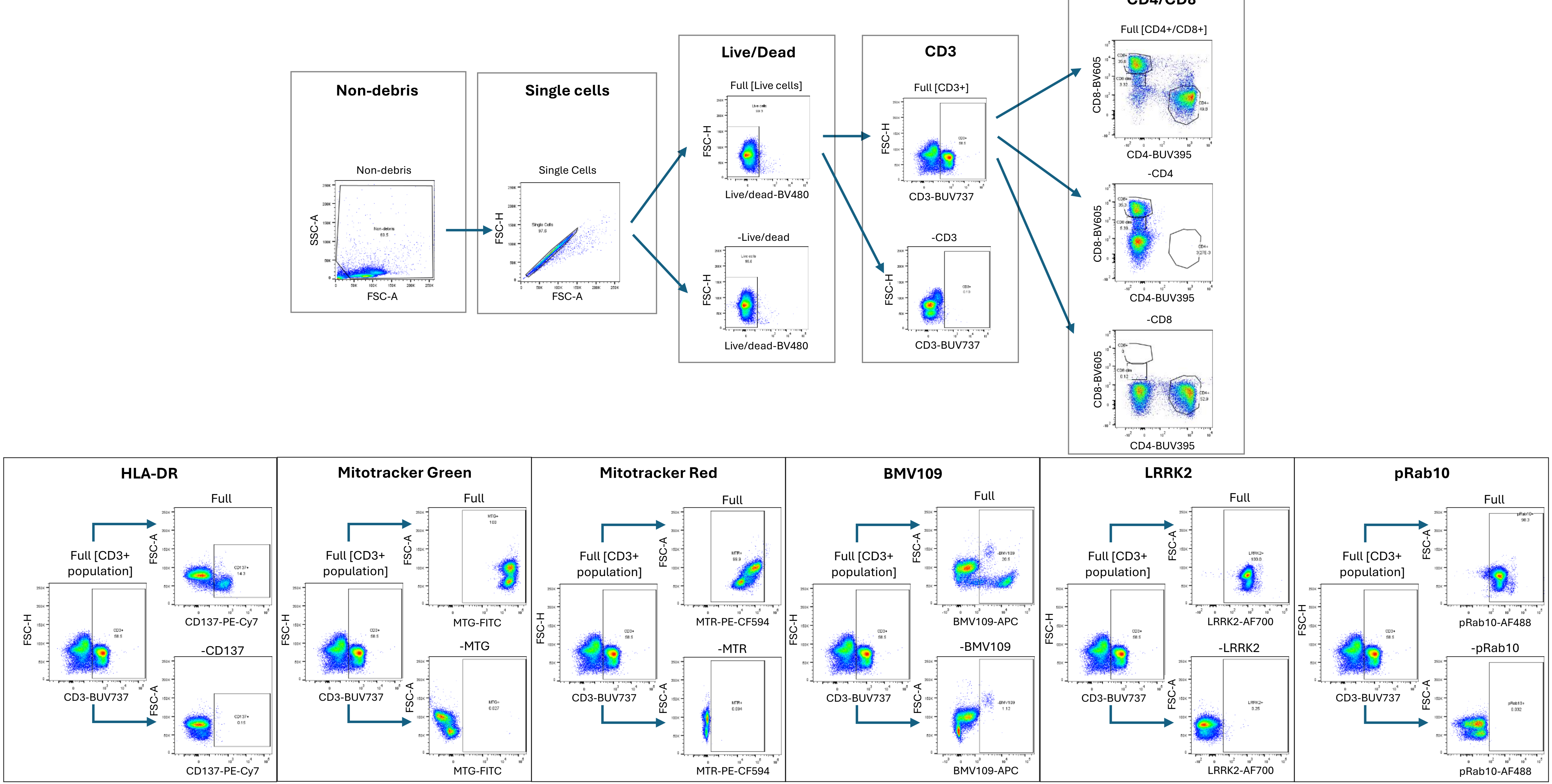

Supplementary Fig. 3: Flow cytometry gating strategy for T cells using fluorescence-minus-one controls. Total PBMCs were stained with antibody-fluorophore conjugates and gates were defined for positive and negative populations based on <1% negatively stained cells in FMOC.

Supplementary Fig. 4: Flow cytometry gating strategy for monocytes using fluorescence-minus-one controls

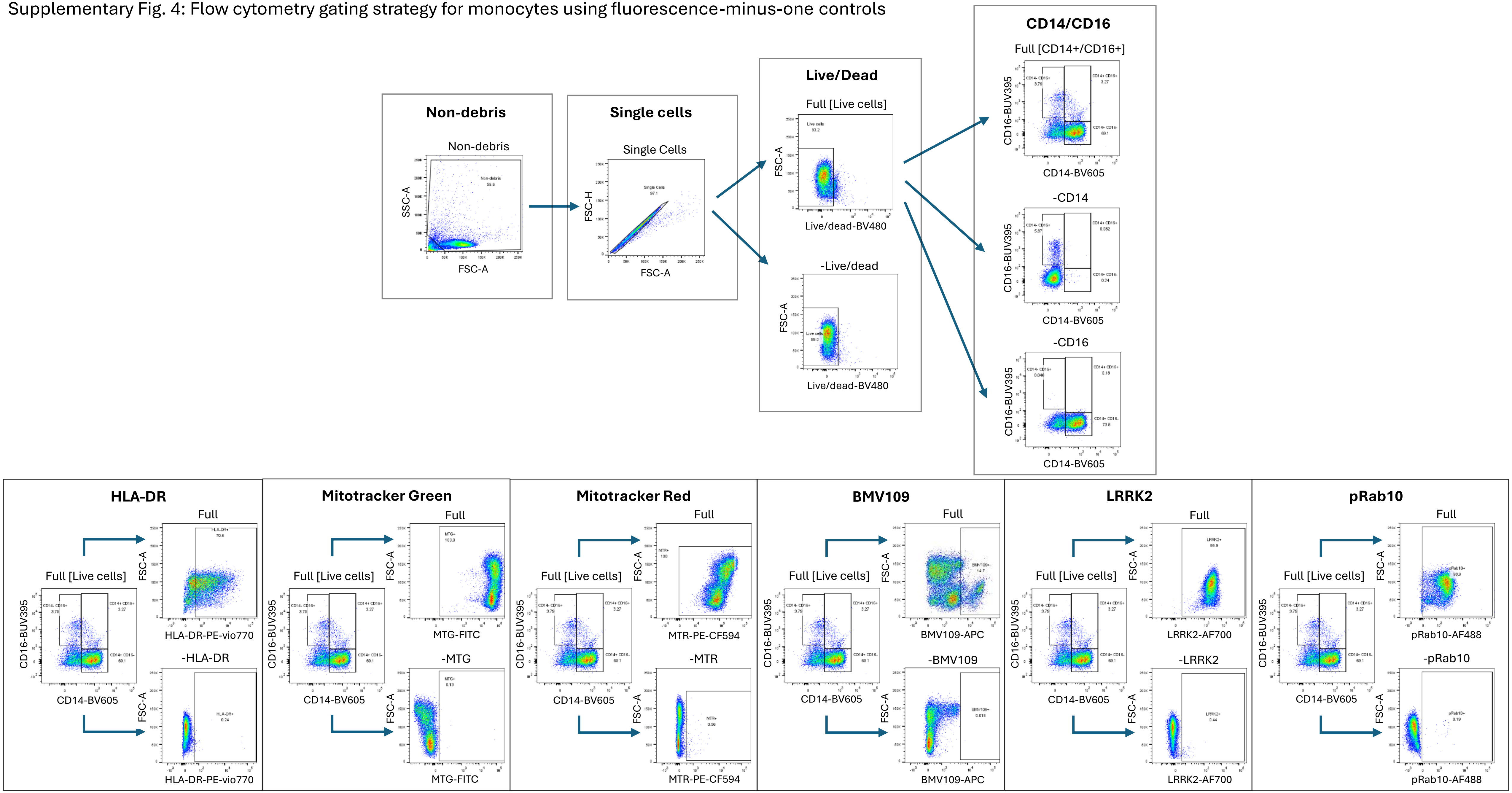

Supplementary Fig. 4: Flow cytometry gating strategy for monocytes using fluorescence-minus-one controls. Isolated monocytes were stained with antibody-fluorophore conjugates and gates were defined for positive and negative populations based on <1% negatively stained cells in FMOC.

Supplementary Fig. 5: PBMC subtype counts

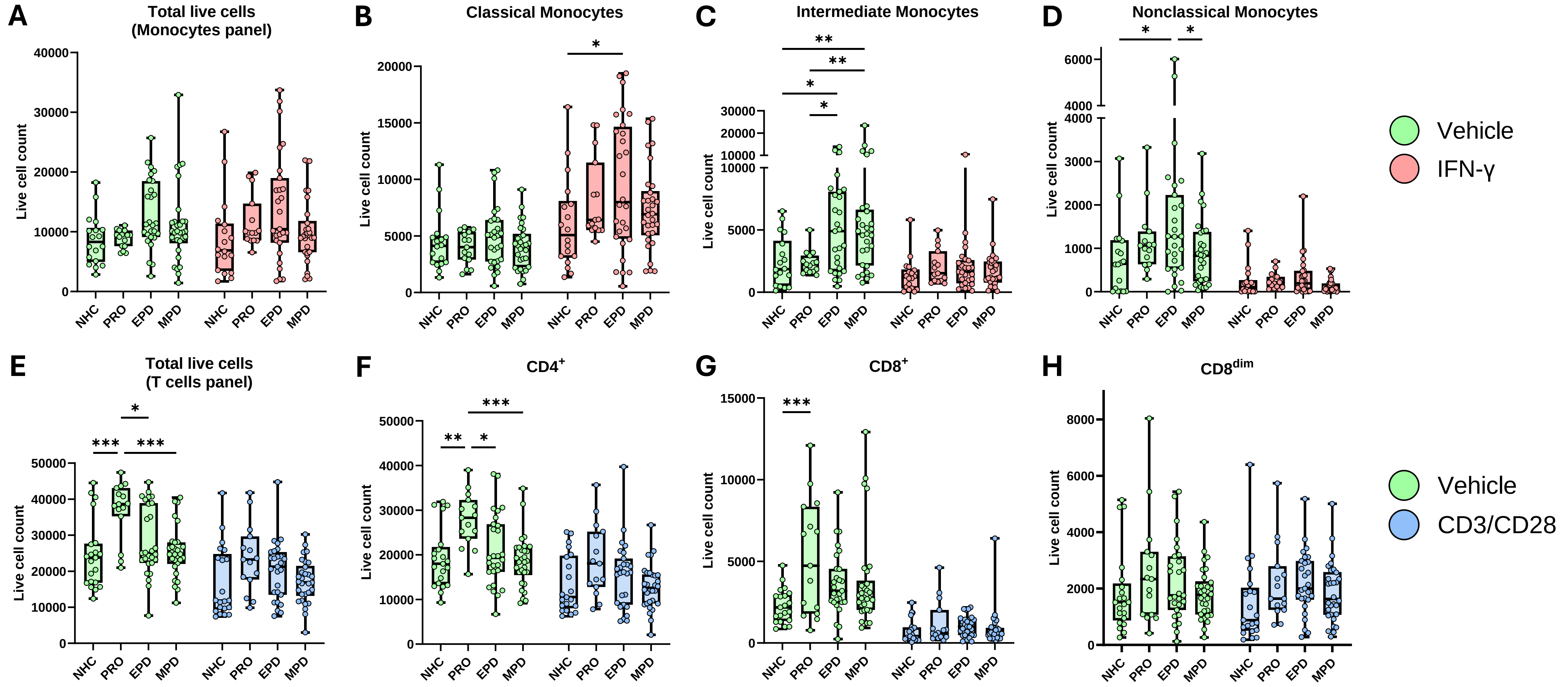

Supplementary Fig. 5: PBMC subtype counts. Box plots depicting the raw counts of live monocytes and T cells obtained via flow cytometry for vehicle treated and stimulated cells obtained from NHCs, prodromal PD patients, EPD patients, and MPD patients. **A** Raw counts of total monocytes ($CD14^+$ and/or $CD16^+$) among live cells. **B** Raw counts of classical monocytes ($CD14^+CD16^-$) among total monocytes. **C** Raw counts of intermediate monocytes ($CD14^+CD16^+$) among total monocytes. **D** Raw counts of nonclassical monocytes ($CD14^{dim}CD16^+$) among total monocytes. **E** Raw counts of total $CD3^+$ T cells among total live cells. **F** Raw counts of $CD4^+CD8^-$ among total $CD3^+$ T lymphocytes. **G** Raw counts of $CD4^-CD8^+$ among total $CD3^+$ T lymphocytes. **H** Raw counts of $CD4^-CD8^{dim}$ among total $CD3^+$ T lymphocytes. Box plots show individual values, median and interquartile range (box), and minimum-maximum range (whiskers). NHC neurologically healthy controls, $n = 21$ biologically independent samples; PRO patients with prodromal PD, $n = 15$ biologically independent samples; EPD patients with early-stage PD, $n = 27$ biologically independent samples; MPD patients with moderate-stage PD, $n = 30$ biologically independent samples. Each symbol represents the measurement from a single individual. The results in **A-H** were analyzed using two-way ANOVA with Tukey's corrections for multiple comparisons. Only within treatment comparisons are shown. Statistical significance is denoted by asterisks (* $p < 0.05$, ** $p < 0.01$, *** $p < 0.001$). Not all samples were run to completion through the cytometer, therefore these counts do not represent the total number of cells plated.

Supplementary Fig. 6: Monocyte mitochondrial health across multiple stages of PD

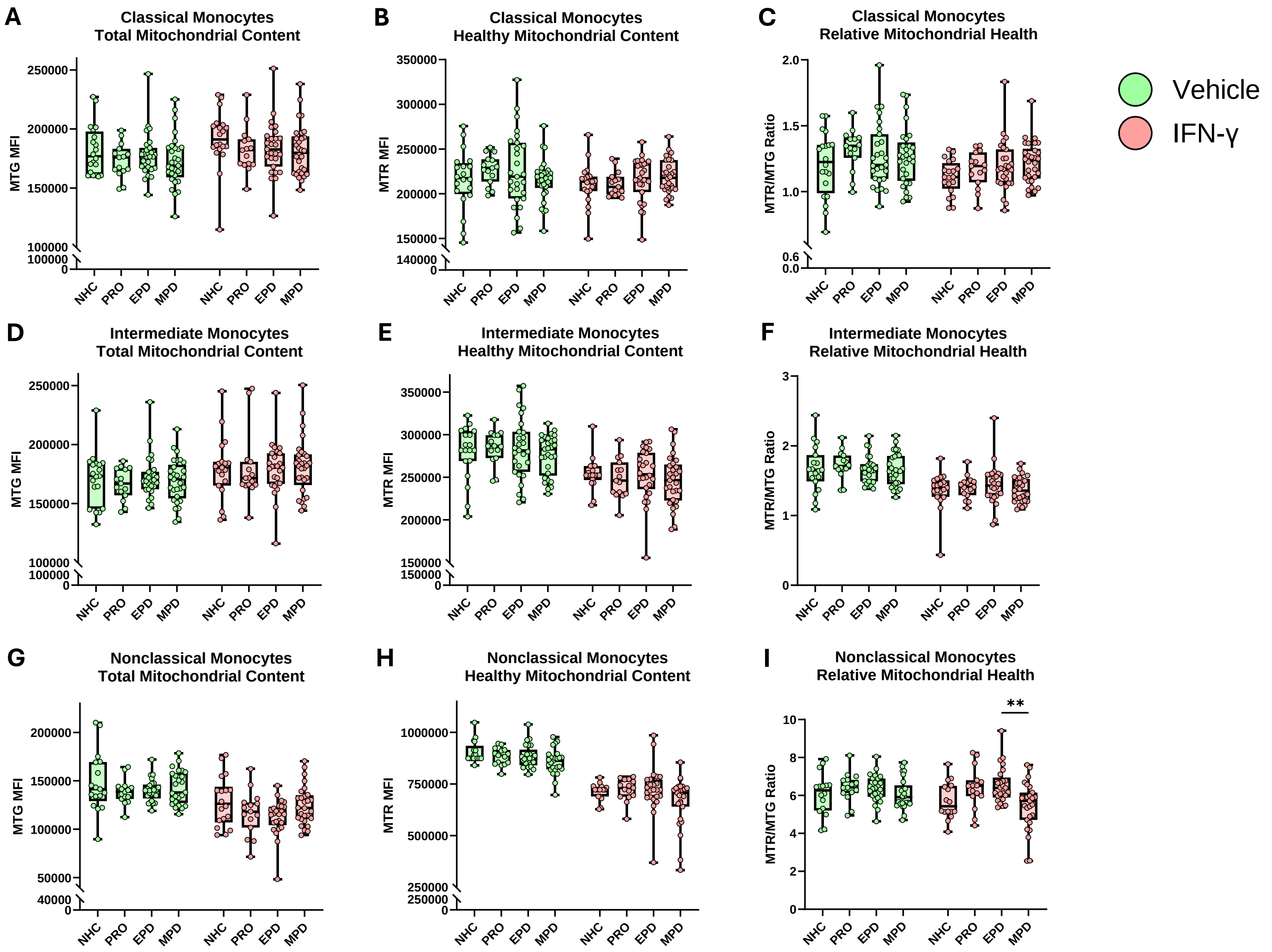

Supplementary Fig. 6: Monocyte mitochondrial health across multiple stages of PD. Box plots depicting the mitochondrial content and mitochondrial health after immune stimulation of monocyte subsets from NHCs, prodromal PD patients, EPD patients, and MPD patients. **A** Total mitochondrial content of classical monocytes (CD14^{+}CD16^{-}). **B** Healthy mitochondrial content with negative membrane potential in classical monocytes (CD14^{+}CD16^{-}). **C** Ratio of healthy mitochondrial content divided by the total in classical monocytes (CD14^{+}CD16^{-}). **D** Total mitochondrial content of intermediate monocytes (CD14^{+}CD16^{+}). **E** Healthy mitochondrial content with negative membrane potential in intermediate monocytes (CD14^{+}CD16^{+}). **F** Ratio of healthy mitochondrial content divided by the total in intermediate monocytes (CD14^{+}CD16^{+}). **G** Total mitochondrial content of nonclassical monocytes (CD14^{dim}CD16^{+}). **H** Healthy mitochondrial content with negative membrane potential in nonclassical monocytes (CD14^{dim}CD16^{+}). **I** Ratio of healthy mitochondrial content divided by the total in nonclassical monocytes (CD14^{dim}CD16^{+}). Box plots show individual values, median and interquartile range (box), and minimum-maximum range (whiskers). NHC neurologically healthy controls, n = 21 biologically independent samples; PRO patients with prodromal PD, n = 15 biologically independent samples; EPD patients with early-stage PD, n = 27 biologically independent samples; MPD patients with moderate-stage PD, n = 30 biologically independent samples. Each symbol represents the measurement from a single individual. The results in **A-I** were analyzed using two-way ANOVA with Tukey's corrections for multiple comparisons. Only within treatment comparisons are shown. Statistical significance is denoted by asterisks (* $p < 0.05$, ** $p < 0.01$, *** $p < 0.001$). MTG MitoTracker Green FM, MTR MitoTracker Red CMXRos, MFI median fluorescence intensity.

Supplementary Fig. 7: Motor impairment is not significantly correlated with T cell stimulation-dependent cytokine secretion

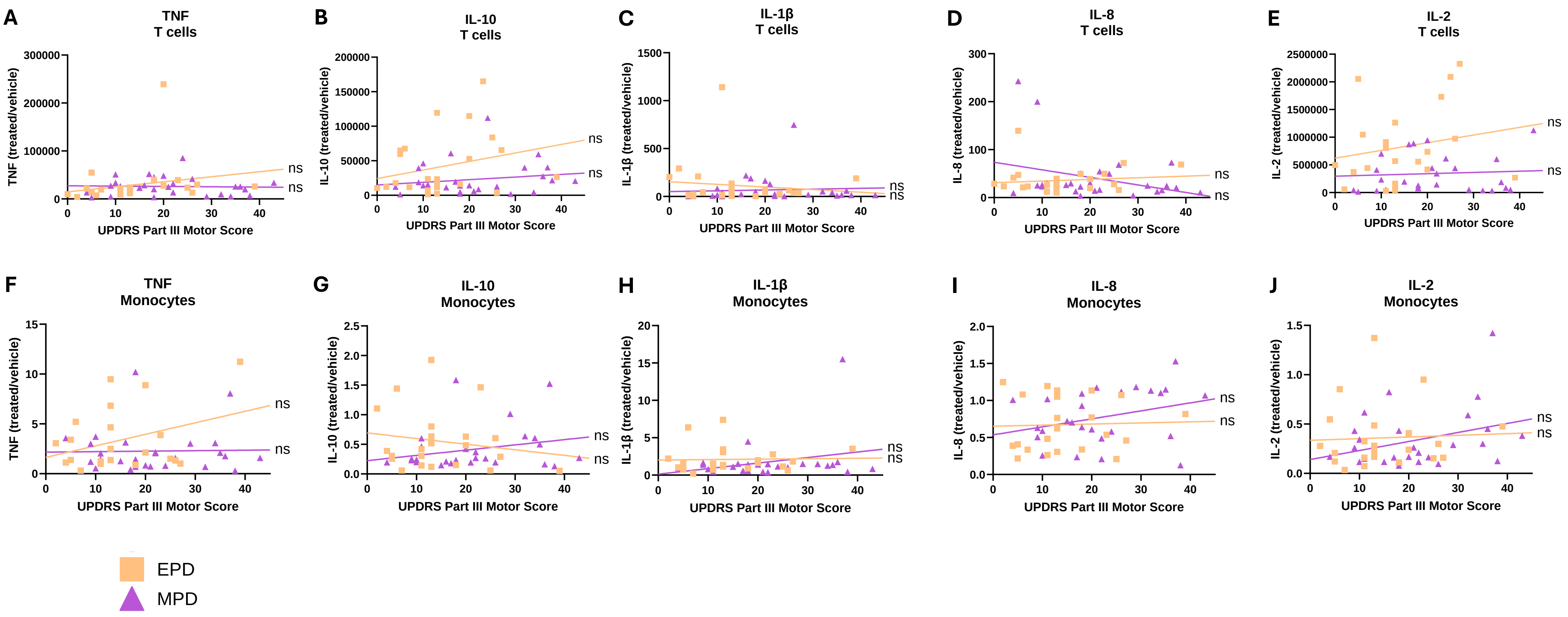

Supplementary Fig. 7: Motor impairment is not significantly correlated with T cell stimulation-dependent cytokine secretion. Linear regression was used to plot the relationship between UPDRS scores from early and moderate stage PD patients and stimulation-dependent cytokine secretion. (**A**) TNF secretion from T cells [(EPD: p = 0.323, R^2 = 0.0444), (MPD: p = 0.841, R^2 = 0.002)]. (**B**) IL-10 secretion from T cells [(EPD: p = 0.212, R^2 = 0.070), (MPD: p = 0.379, R^2 = 0.030)]. (**C**) IL-1β secretion from T cells [(EPD: p = 0.610, R^2 = 0.012), (MPD: p = 0.747, R^2 = 0.004)]. (**D**) IL-8 secretion from T cells [(EPD: p = 0.595, R^2 = 0.013), (MPD: p = 0.101, R^2 = 0.100)]. (**E**) IL-2 secretion from T cells [(EPD: p = 0.412, R^2 = 0.031), (MPD: p = 0.7158, R^2 = 0.005)]. (**F**) TNF secretion from monocytes [(EPD: p = 0.123, R^2 = 0.115), (MPD: p = 0.909, R^2 = 0.001)]. (**G**) IL-10 secretion from monocytes [(EPD: p = 0.448, R^2 = 0.029), (MPD: p = 0.217, R^2 = 0.060)]. (**H**) IL-1β secretion from monocytes [(EPD: p = 0.895, R^2 = 0.001), (MPD: p = 0.164, R^2 = 0.076)]. (**I**) IL-8 secretion from monocytes [(EPD: p = 0.869, R^2 = 0.001), (MPD: p = 0.109, R^2 = 0.100)]. (**J**) IL-2 secretion from monocytes [(EPD: p =0.834, R^2 = 0.002), (MPD: p = 0.098, R^2 = 0.106)]. *p*-value based on Pearson correlation. early-stage PD, n = 23 biologically independent samples; moderate-stage PD, n = 27 biologically independent samples. Each symbol represents the measurement from a single individual. * signifies that the slope of the line is significantly different from zero ($p < 0.05$). *ns* signifies that the slope of the line is not significantly different from zero ($p > 0.05$).

Supplementary Fig. 8: Lysosomal content of monocyte and T cell subsets across multiple stages of PD

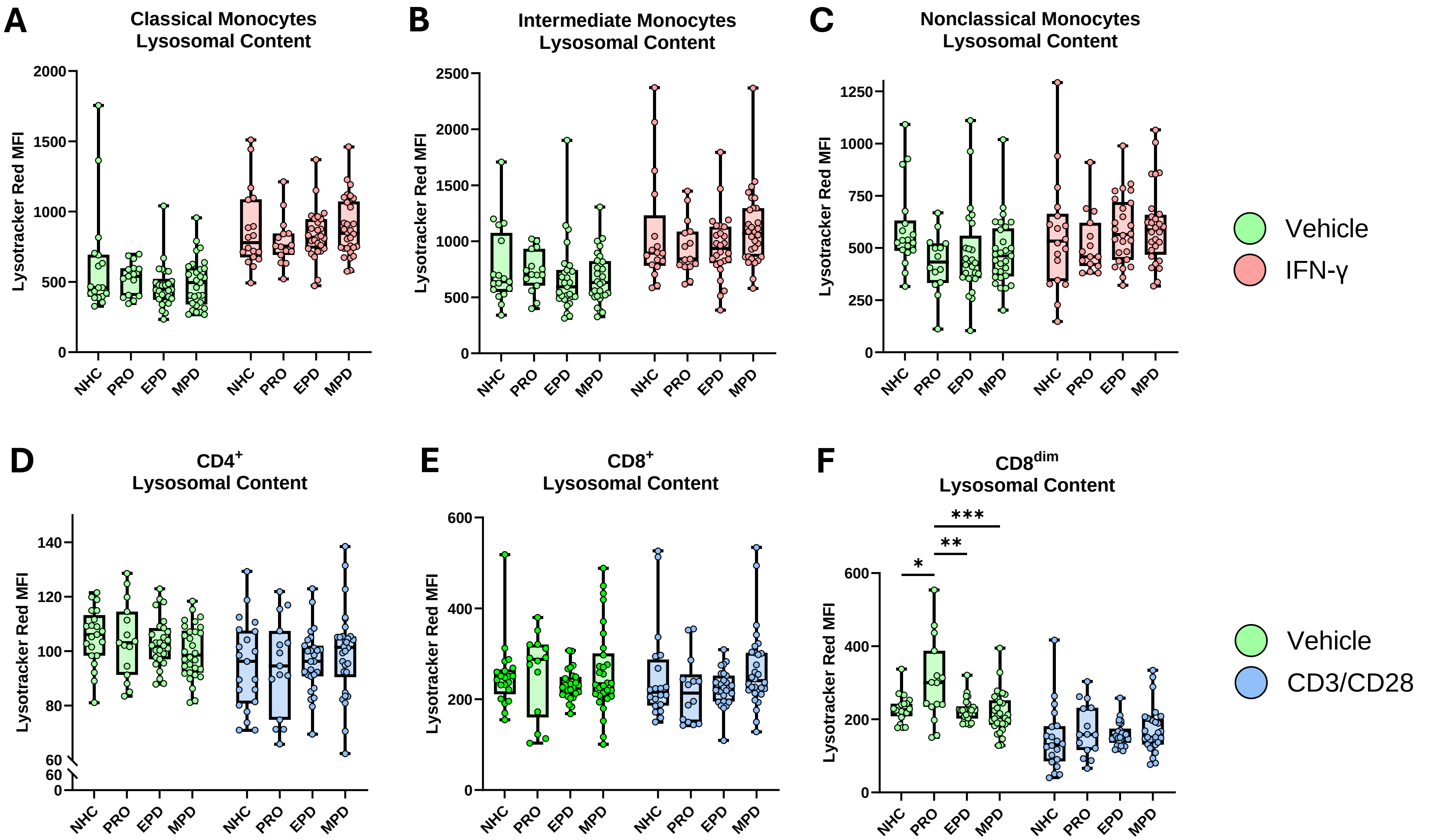

Supplementary Fig. 8: Lysosomal content of monocyte and T cell subsets across multiple stages of PD. Box plots depicting the lysosomal content after immune stimulation of monocyte and T cell subsets from NHCs, prodromal PD patients, EPD patients, and MPD patients. Lysosomal content was quantified using MFI of Lysotracker Red DND. **A** Total lysosomal content of classical monocytes ($CD14^{+}CD16^{-}$). **B** Total lysosomal content of intermediate monocytes ($CD14^{+}CD16^{+}$). **C** Total lysosomal content of nonclassical monocytes ($CD14^{dim}CD16^{+}$). **D** Total lysosomal content of $CD4^{+}/CD8^{-}$ T cells. **E** Total lysosomal content of $CD4^{-}/CD8^{+}$ T cells. **H** Total lysosomal content of $CD4^{-}/CD8^{dim}$ T cells. Box plots show individual values, median and interquartile range (box), and minimum-maximum range (whiskers). NHC neurologically healthy controls, *n* = 21 biologically independent samples; PRO patients with prodromal PD, *n* = 15 biologically independent samples; EPD patients with early-stage PD, *n* = 27 biologically independent samples; MPD patients with moderate-stage PD, *n* = 30 biologically independent samples. Each symbol represents the measurement from a single individual. The results in **A-F** were analyzed using two-way ANOVA with Tukey's corrections for multiple comparisons. Only within treatment comparisons are shown. Statistical significance is denoted by asterisks (* $p < 0.05$, ** $p < 0.01$, *** $p < 0.001$). MFI median fluorescence intensity.

Supplementary Fig. 9: Pan-cathepsin activity in T cell subsets after stimulation

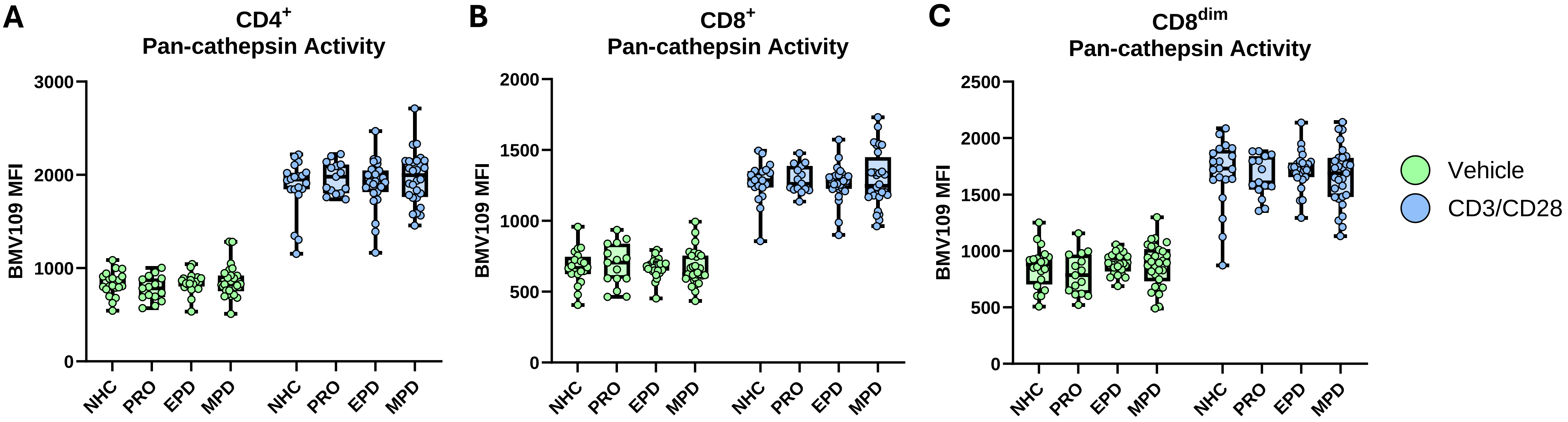

Supplementary Fig. 9: Pan-cathepsin activity in T cell subsets. Box plots depicting the pan-cathepsin activity after CD3/CD28 Dynabead stimulation of T cell subsets from NHCs, prodromal PD patients, EPD patients, and MPD patients. Pan-cathepsin activity was quantified using MFI of BMV109. **A** Pan-cathepsin activity of $CD4^+/CD8^-$ T cells. **B** Pan-cathepsin activity of $CD4^-/CD8^+$ T cells. **C** Pan-cathepsin activity of $CD4^-/CD8^{dim}$ T cells. Box plots show individual values, median and interquartile range (box), and minimum-maximum range (whiskers). NHC neurologically healthy controls, n = 21 biologically independent samples; PRO patients with prodromal PD, n = 15 biologically independent samples; EPD patients with early-stage PD, n = 27 biologically independent samples; MPD patients with moderate-stage PD, n = 30 biologically independent samples. Each symbol represents the measurement from a single individual. The results in **A-C** were analyzed using two-way ANOVA with Tukey's corrections for multiple comparisons. Only within treatment comparisons are shown. Statistical significance is denoted by asterisks (* $p < 0.05$, ** $p < 0.01$, *** $p < 0.001$). MFI median fluorescence intensity.

Supplementary Fig. 10: LRRK2 expression and pRab10 expression in monocyte subsets

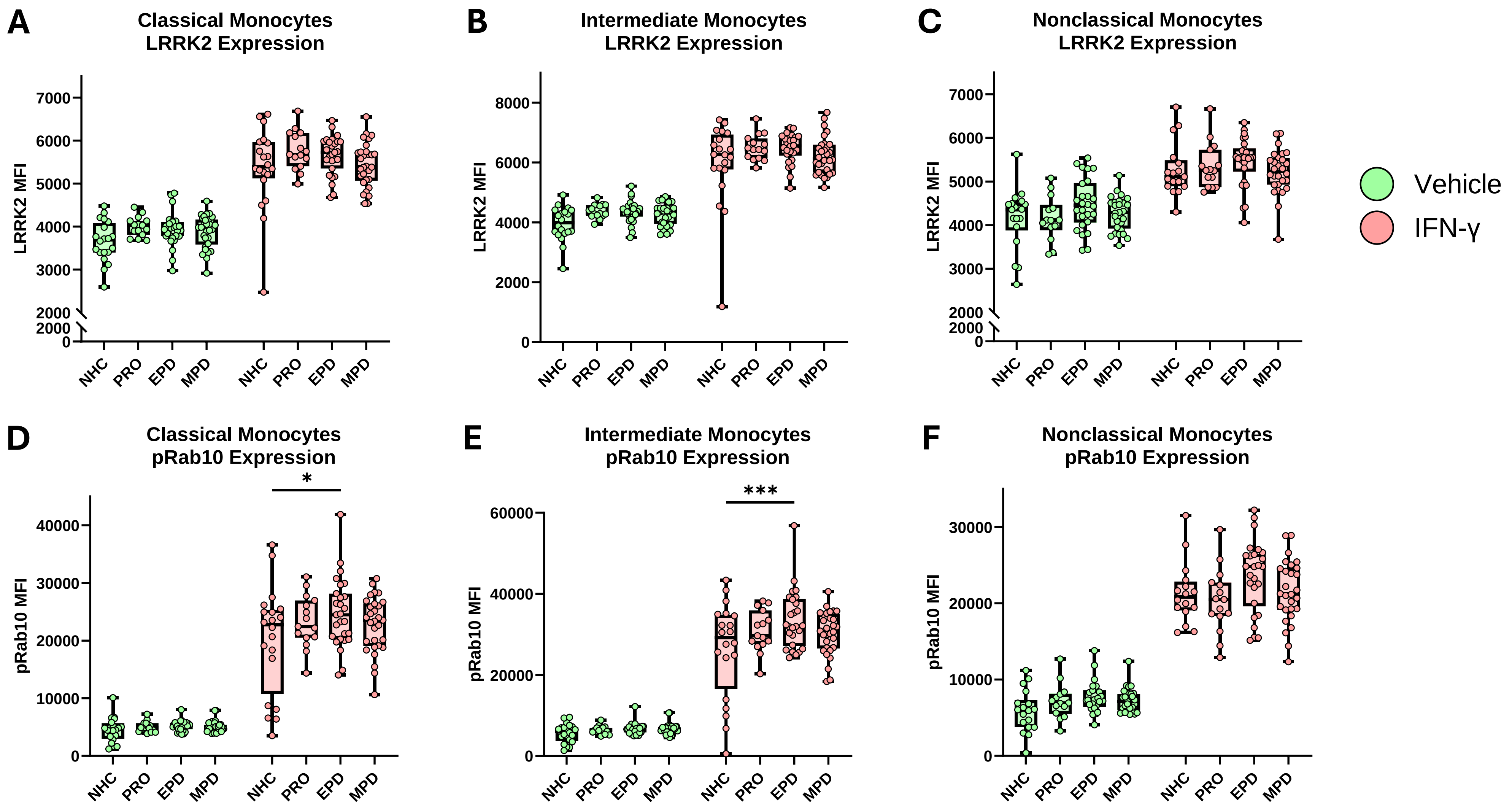

Supplementary Fig. 10: LRRK2 expression and pRab10 expression in monocyte subsets. Box plots depicting the expression of LRRK2 and pRab10 after IFNγ stimulation of monocyte subsets from NHCs, prodromal PD patients, EPD patients, and MPD patients. **A** LRRK2 expression in classical monocytes ($CD14^{+}CD16^{-}$). **B** LRRK2 expression in intermediate monocytes ($CD14^{+}CD16^{+}$). **C** LRRK2 expression in nonclassical monocytes ($CD14^{dim}CD16^{+}$). **D** pRab10 expression in classical monocytes ($CD14^{+}CD16^{-}$). **E** pRab10 expression in intermediate monocytes ($CD14^{+}CD16^{+}$). **F** pRab10 expression in nonclassical monocytes ($CD14^{dim}CD16^{+}$). Box plots show individual values, median and interquartile range (box), and minimum-maximum range (whiskers). NHC neurologically healthy controls, n = 21 biologically independent samples; PRO patients with prodromal PD, n = 15 biologically independent samples; EPD patients with early-stage PD, n = 27 biologically independent samples; MPD patients with moderate-stage PD, n = 30 biologically independent samples. Each symbol represents the measurement from a single individual. The results in **A-F** were analyzed using two-way ANOVA with Tukey's corrections for multiple comparisons. Only within treatment comparisons are shown. Statistical significance is denoted by asterisks (* $p < 0.05$, ** $p < 0.01$, *** $p < 0.001$). MFI median fluorescence intensity.

Supplementary Fig. 11: LRRK2 expression and pRab10 expression in T cell subsets

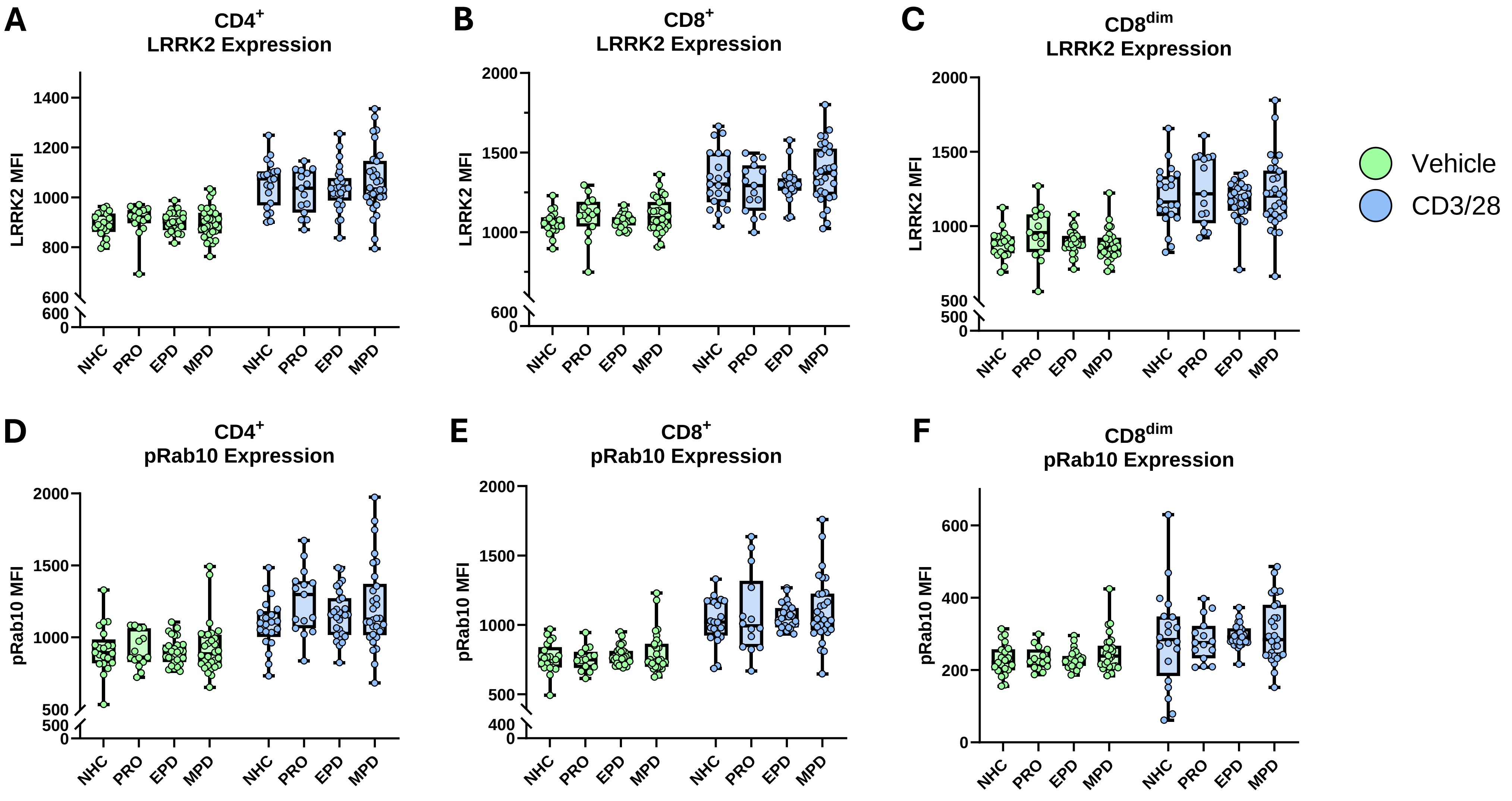

Supplementary Fig. 11: LRRK2 expression and pRab10 expression in T cell subsets. Box plots depicting the expression of LRRK2 and pRab10 after CD3/CD28 stimulation of T cells from NHCs, prodromal PD patients, EPD patients, and MPD patients. **A** LRRK2 expression in $CD4^+$/$CD8^-$ T cells. **B** LRRK2 expression in $CD4^-$/$CD8^+$ T cells. **C** LRRK2 expression in $CD4^-$/$CD8^{dim}$ T cells. **D** pRab10 expression in $CD4^+$/$CD8^-$ T cells. **E** pRab10 expression in $CD4^-$/$CD8^+$ T cells. **F** pRab10 expression in $CD4^-$/$CD8^{dim}$ T cells. Box plots show individual values, median and interquartile range (box), and minimum-maximum range (whiskers). NHC neurologically healthy controls, $n = 21$ biologically independent samples; PRO patients with prodromal PD, $n = 15$ biologically independent samples; EPD patients with early-stage PD, $n = 27$ biologically independent samples; MPD patients with moderate-stage PD, $n = 30$ biologically independent samples. Each symbol represents the measurement from a single individual. The results in **A-F** were analyzed using two-way ANOVA with Tukey's corrections for multiple comparisons. Only within treatment comparisons are shown. Statistical significance is denoted by asterisks (* $p < 0.05$). MFI median fluorescence intensity.

Supplementary Table 1: Flow cytometry T cell marker antibody panel

Flow cytometry T cell marker antibody panel				
Target	Conjugate	Catalog #	Dilution	Company/Manufacturer
CD3	BUV737	BDB612752	1:50	BD Biosciences
CD4	BUV395	BDB564724	1:50	BD Biosciences
CD8	BV605	BDB564116	1:50	BD Biosciences
CD137	Pe-Cy7	309818	1:20	BioLegend
FcR block	-	422301	1:20	BioLegend
Live/dead Violet	-	L34962	1:2000	Invitrogen

Supplementary Table 1: Flow cytometry T cell marker antibody panel. This antibody panel was used for both unfixed and fixed cell panels to identify cell-surface markers of T cell subsets.

Supplementary Table 2: Flow cytometry monocyte marker antibody panel

Flow cytometry monocyte marker antibody panel				
Target	Conjugate	Catalog #	Dilution	Company/Manufacturer
CD14	BV605	BDB564054	1:25	BD Biosciences
CD16	BUV395	BDB563785	1:50	BD Biosciences
HLA-DR	PE-vio770	130-113-403	1:100	Miltenyi Biotec
FcR block	-	422301	1:20	BioLegend
Live/dead Violet	-	L34962	1:2000	Invitrogen

Supplementary Table 2: Flow cytometry monocyte marker antibody panel. This antibody panel was used for both unfixed and fixed cell panels to identify cell-surface markers of monocyte subsets.
