# Supplementary material for: Peripheral immune cell response to stimulation stratifies Parkinson’s disease progression from prodromal to clinical stages
Source: Commun Biol. 2025 May 8;8:716. doi: 10.1038/s42003-025-08088-7 (PMC12062209; doi:10.1038/s42003-025-08088-7)
Supplement: Supplementary file 2 — Description of Additional Supplementary Files [file 42003_2025_8088_MOESM2_ESM.pdf]

Description of Additional Supplementary Files

File Name: Supplementary Data 1
Description: This file contains additional clinical information on the participant cohorts used in this study.

File Name: Supplementary Data 2
Description: The source data behind the graphs in the paper from flow cytometry and cytokine secretion.
