# Supplementary material for: Peripheral immune cell response to stimulation stratifies Parkinson’s disease progression from prodromal to clinical stages
Source: Commun Biol. 2025 May 8;8:716. doi: 10.1038/s42003-025-08088-7 (PMC12062209; doi:10.1038/s42003-025-08088-7)
Supplement: Supplementary file 5 — Reporting Summary [file 42003_2025_8088_MOESM5_ESM.pdf]

nature portfolio

Corresponding author(s): Rebecca L. Wallings and Malú Gámez Tansey

Last updated by author(s): Mar 27, 2025

Reporting Summary

Nature Portfolio wishes to improve the reproducibility of the work that we publish. This form provides structure for consistency and transparency in reporting. For further information on Nature Portfolio policies, see our Editorial Policies and the Editorial Policy Checklist.

Statistics

For all statistical analyses, confirm that the following items are present in the figure legend, table legend, main text, or Methods section.

n/a	Confirmed	
☐	☒	The exact sample size (*n*) for each experimental group/condition, given as a discrete number and unit of measurement
☐	☒	A statement on whether measurements were taken from distinct samples or whether the same sample was measured repeatedly
☐	☒	The statistical test(s) used AND whether they are one- or two-sided<br>*Only common tests should be described solely by name; describe more complex techniques in the Methods section.*
☐	☒	A description of all covariates tested
☐	☒	A description of any assumptions or corrections, such as tests of normality and adjustment for multiple comparisons
☐	☒	A full description of the statistical parameters including central tendency (e.g. means) or other basic estimates (e.g. regression coefficient) AND variation (e.g. standard deviation) or associated estimates of uncertainty (e.g. confidence intervals)
☐	☒	For null hypothesis testing, the test statistic (e.g. *F*, *t*, *r*) with confidence intervals, effect sizes, degrees of freedom and *P* value noted<br>*Give P values as exact values whenever suitable.*
☒	☐	For Bayesian analysis, information on the choice of priors and Markov chain Monte Carlo settings
☒	☐	For hierarchical and complex designs, identification of the appropriate level for tests and full reporting of outcomes
☐	☒	Estimates of effect sizes (e.g. Cohen's *d*, Pearson's *r*), indicating how they were calculated

Our web collection on statistics for biologists contains articles on many of the points above.

Software and code

Policy information about availability of computer code

Data collection	Flow cytometry data were acquired using the BD FACSDiva™ Software (v9.3.1). Cytokine concentration data were acquired using Discovery Workbench 4.0 Software from Meso Scale Discovery.
Data analysis	Flow cytometry data were analyzed using FlowJo v10.10.0. The MSD cytokine analysis was performed using the Discovery Workbench v 4.0 software. All the statistical analysis including the correlation analysis was performed using GraphPad Prism v10. BioRender was used to create illustrations for figures.

For manuscripts utilizing custom algorithms or software that are central to the research but not yet described in published literature, software must be made available to editors and reviewers. We strongly encourage code deposition in a community repository (e.g. GitHub). See the Nature Portfolio guidelines for submitting code & software for further information.

Data

Policy information about availability of data

All manuscripts must include a data availability statement. This statement should provide the following information, where applicable:

- Accession codes, unique identifiers, or web links for publicly available datasets
- A description of any restrictions on data availability
- For clinical datasets or third party data, please ensure that the statement adheres to our policy

All data on the measured variables relating to immune cell function and cytokine secretion in relation to Parkinson’s disease status and progression that support the

findings of this study are included within this paper and its Supplementary Information and Supplementary Data files. Source data are provided with this paper. Only the relevant clinical data (i.e., UPDRS scores, disease duration) that were discussed in the article are provided in Source Data.

Research involving human participants, their data, or biological material

Policy information about studies with human participants or human data. See also policy information about sex, gender (identity/presentation), and sexual orientation and race, ethnicity and racism.

Reporting on sex and gender

During patient recruitment, sex was considered in the study design to ensure that groups would be sex-matched. We performed statistical analysis to demonstrate that the proportions of males/females in each group were not statistically different, and these results are shown in the Supplementary Information. Sex-specific analysis was not performed. Sex was determined based on self-reporting, supported by medical records, and all the selected participants were examined by trained neurologists to ensure accuracy. The overall numbers recruited are as follows: neurologically healthy controls (NHC) (10M, 11F), REM sleep behavior disorder (RBD) (8M, 7F), early PD (14M, 13F), and moderate PD (16M, 15F).

Reporting on race, ethnicity, or other socially relevant groupings

N/A

Population characteristics

We recruited participants from the UF Norman Fixel Institute for Neurological Diseases and controlled for several confounding factors including age, sex, medications, smoking history, NSAID usage, and comorbidities. Genetic testing was performed to rule out the LRRK2-G2019S mutation, one of the most common genetic causes of familial PD. Full inclusion/exclusion details are provided in the methods section of the research article. The overall numbers recruited are as follows: NHC (10M, 11F), RBD (8M, 7F), early PD (14M, 13F), and moderate PD (16M, 15F). The average ages for each group were as follows: NHC = 65.29, RBD = 68.93, early PD = 67.19, moderate PD = 66.97. Full demographics and clinical information for the study population are available in Table 1.

Recruitment

We recruited participants from the UF Norman Fixel Institute for Neurological Diseases. The overall selection criteria are provided in the methods section of the research article. Patients were screened based on PD and RBD diagnoses and disease duration. Subjects were excluded based on age (no younger than 30 and no older than 80 years of age), known familial PD mutations and/or other known neurological conditions, chronic or recent infections, or autoimmune comorbidities. There is no obvious recruitment self-selection bias.

Ethics oversight

The study protocol was reviewed and approved the University of Florida Institutional Review Board (IRB202002639). Informed consent was obtained before each participant was recruited into the study.

Note that full information on the approval of the study protocol must also be provided in the manuscript.

Field-specific reporting

Please select the one below that is the best fit for your research. If you are not sure, read the appropriate sections before making your selection.

☒ Life sciences ☐ Behavioural & social sciences ☐ Ecological, evolutionary & environmental sciences

For a reference copy of the document with all sections, see nature.com/documents/nr-reporting-summary-flat.pdf

Life sciences study design

All studies must disclose on these points even when the disclosure is negative.

Sample size

The planned sample size in the study protocol was 30 NHC, 30 RBD, 30 early PD, and 30 moderate PD. Power analysis was conducted using G*Power 3.1.9.7 and indicates that, based on previous data published by our research group quantifying cytokine changes in plated monocytes and T cells from iPD patients and HCs [Cook et al., 2017], in order to be 80% powered 21 individuals per group are required for an effect size of 0.96 with a 0.05 type I error rate in order to see differences in the means of cytokine pg/ml. Recruitment for this study was scheduled to start in 2020, however due to the COVID-19 pandemic, we encountered difficulties reaching the desired recruitment numbers for each group. Following the stringent inclusion and exclusion criteria listed, we were able to recruit 21 NHCs, 15 RBD patients, 27 early PD patients, and 31 moderate PD patients.

Data exclusions

Data were excluded if the data point was identified as an outlier using the determination of being more than 3 standard deviations above or below the mean. For one moderate PD patient, data were unable to be collected for the cytokine concentration analyses because the cell-free media was lost prior to analysis.

Replication

These results are the initial findings, and due to limitations in the number of cryovials of PBMCs collected for each patient, it is not feasible to repeat all of the assays for replication attempts.

Randomization

After patient recruitment, blood collection, and PBMC isolation for all participants was completed, the patient IDs were randomly assigned into batches to be processed. The researcher who performed the random assignment did not take part in performing the experiments.

Blinding

The researchers who conducted the assays were completely blinded to subject groupings. This blinding was maintained throughout collection of all flow cytometry data and cytokine multiplexing assay data. Unblinding was only performed after all data collection had been completed. Unblinding was performed prior to analysis because it was necessary to normalize flow cytometry data across batches to account for day-to-

day fluctuations in equipment functionality (e.g. laser power).

Reporting for specific materials, systems and methods

We require information from authors about some types of materials, experimental systems and methods used in many studies. Here, indicate whether each material, system or method listed is relevant to your study. If you are not sure if a list item applies to your research, read the appropriate section before selecting a response.

Materials & experimental systems

n/a	Involved in the study
☐	☒ Antibodies
☒	☐ Eukaryotic cell lines
☒	☐ Palaeontology and archaeology
☒	☐ Animals and other organisms
☐	☒ Clinical data
☒	☐ Dual use research of concern
☒	☐ Plants

Methods

n/a	Involved in the study
☒	☐ ChIP-seq
☐	☒ Flow cytometry
☒	☐ MRI-based neuroimaging

Antibodies

Antibodies used

The antibodies used for flow cytometry including the manufacturer, catalog number, and dilution are included in Table 2 and Table 3. We provide them here again.
- Ab Target, Fluorochrome, Dilution factor, Manufacturer, Reference/Catalog number, clone, Lot #
- CD3, BUV737, 1:50, BD Biosciences, BDB612752, SK7, 1137540.
- CD4, BUV395, 1:50, BD Biosciences, BDB564724, RPA-T4, 1130442.
- CD8, BV605, 1:50, BD Biosciences, BDB564116, SK1, 1005860.
- CD137, Pe-Cy7, 1:20, BioLegend, 309818, 4B4-1, B393119.
- CD14, BV605, 1:25, BD Biosciences, BDB564054, M5E2, 1004483.
- CD16, BUV395, 1:50, BD Biosciences, BDB563785, 3G8, 3180041.
- HLA-DR, Pe-vio770, 1:100, Miltenyi Biotec, 130-113-403, AC122, 1323110577.
- pRab10 (phospho T73) rabbit monoclonal primary antibody, N/A, 1:200, Abcam, ab241060, 1054724-5.
- Donkey anti-Rabbit IgG antibody, AF488, 1:1000, Invitrogen, A21206, 2873188.
- LRRK2, Alexa Fluor 700, 1:100, Novus Biologicals, NB300-268AF700, D167053.
- FcR block, N/A, 1:20, BioLegend, 422301, B426260.

Details for additional fluorescent probes:
- Live/dead fixable Violet, 1:2000, Invitrogen, L34962, 2725316.
- MitoTracker Red CMXRos, 1 µM, Invitrogen, M7512, 2351994.
- MitoTracker Green FM, 1 µM, Invitrogen, M7514, 2420614.
- LysoTracker Red DND-99, 200 nM / 500 nM for monocytes/T cells respectively, Invitrogen, L7528, 30D1-1.
- BMV109 Pan Cathepsin Probe, 1 µM, Vergent Biosciences, 40200-200.

Validation

The reaction species and the applications (specifically flow cytometry) of the antibodies used in this study have been validated by the commercial manufacturers, and this information is directly stated in the datasheets provided by the manufacturers.

The general routine validation statements for flow cytometry analysis are also available on the website of the corresponding provider. For BD, please visit: https://www.bdbiosciences.com/en-lu/products/reagents/flow-cytometry-reagents/research-reagents/quality-and-reproducibility; For Biolegend, please visit: https://www.biolegend.com/en-us/quality/quality-control; For the one antibody we used from Abcam, please visit: https://www.abcam.com/en-us/products/primary-antibodies/rab10-phospho-t73-antibody-mjf-r21-22-5-ab241060; For the one antibody we used from Novus Biologicals, please visit: https://www.novusbio.com/products/lrrk2-antibody_nb300-268af700; For Miltenyi Biotec, please visit: https://www.miltenyibiotec.com/US-en/products/macs-antibodies/antibody-validation.html.

Clinical data

Policy information about clinical studies
All manuscripts should comply with the ICMJE guidelines for publication of clinical research and a completed CONSORT checklist must be included with all submissions.

Clinical trial registration

N/A

Study protocol

The full study protocol including updated amendments is provided with the manuscript submission.

Data collection

All participants were recruited at the Norman Fixel Institute for Neurological Diseases at the University of Florida (UF). The first enrolled participant provided blood samples in May 2021, and the last participant's samples were collected in March 2023.

Outcomes

The major expected outcomes were defined and assessed as follows:
- We expected monocytes and T cells from early and moderate iPD patients to exhibit altered stimulation-dependent inflammatory responses (cytokine release, activation markers on immune cells, metabolic organelle function) relative to HCs and prodromal patients resulting from disease-associated changes. We also predicted monocytes and T cells from prodromal patients to represent an 'intermediate' pro-inflammatory state between those from HCs and iPD patients due to the fact that immune cell activation should be an early response to neuronal dysfunction and prior to neurodegeneration. Furthermore, we expected early iPD 'de novo' patients to exhibit greater immune cell dysfunction relative to prodromal patients, with moderate iPD patients exhibiting the most severe cases of immune cell dysfunction; thus placing patients on a spectrum of immune cell dysfunction from prodromal and early iPD, to moderate iPD. We expected changes in cytokine-release to also be accompanied by alterations in lysosomal function such as decreased lysosomal pH and protein degradation. We expected isolated monocytes and T cells studies to be more informative than studies involving mixed cell populations in PBMCs, but given that most biorepositories have primarily PBMCs, there would be significant value in comparing these cell-type specific results to those for total PBMCs reported by other groups.

Plants

Seed stocks

Report on the source of all seed stocks or other plant material used. If applicable, state the seed stock centre and catalogue number. If plant specimens were collected from the field, describe the collection location, date and sampling procedures.

Novel plant genotypes

Describe the methods by which all novel plant genotypes were produced. This includes those generated by transgenic approaches, gene editing, chemical/radiation-based mutagenesis and hybridization. For transgenic lines, describe the transformation method, the number of independent lines analyzed and the generation upon which experiments were performed. For gene-edited lines, describe the editor used, the endogenous sequence targeted for editing, the targeting guide RNA sequence (if applicable) and how the editor was applied.

Authentication

Describe any authentication procedures for each seed stock used or novel genotype generated. Describe any experiments used to assess the effect of a mutation and, where applicable, how potential secondary effects (e.g. second site T-DNA insertions, mosiacism, off-target gene editing) were examined.

Flow Cytometry

Plots

Confirm that:

☒ The axis labels state the marker and fluorochrome used (e.g. CD4-FITC).

☒ The axis scales are clearly visible. Include numbers along axes only for bottom left plot of group (a 'group' is an analysis of identical markers).

☒ All plots are contour plots with outliers or pseudocolor plots.

☒ A numerical value for number of cells or percentage (with statistics) is provided.

Methodology

Sample preparation

- PBMC isolation was accomplished using BD Vacutainer CPT Cell Preparation Tube with Sodium Citrate (BD Biosciences, 362761). Approximately 6 CPT tubes, each containing 8 mL of blood, were collected from each participant. CPT tubes were inverted 8–10 times and centrifuged at room temperature at 1500 x g for 20 min at room temperature. The PBMC enriched layer was transferred to a new 50 mL conical tube and MACS buffer (PBS, 0.5% bovine serum albumin, 20 mM EDTA, pH 7.2) was added to a final volume of 50 mL, followed by centrifugation at 1800 x g for 10 min at room temperature. Following removal of the supernatant, PBMCs were resuspended in 10 mL MACS buffer and counted on a hemocytometer using Trypan blue (1:20 dilution) exclusion to ascertain viability.
- Next, to cryopreserve the samples, PBMCs were centrifuged for 5 min 1800 x g at room temperature. Supernatant was aspirated and cell pellets were gently resuspended in cryopreservation media (54% RPMI 1640, 36% FBS, 10% DMSO) at a final concentration of 10 million cells/mL in cryovials (Simport, T311-2). Cryovials were placed in a room-temperature Mr. Frosty freezing container with isopropanol as per manufacturer's instructions and stored at −80°C overnight. After overnight storage at −80°C, the next day cryovials were removed from freezing containers and placed into liquid nitrogen for long-term storage.
- For cryorecovery, cryovials of PBMCs were retrieved from liquid nitrogen, rapidly thawed in a water bath at 37°C, and rapidly added to 25 mL of 37°C filter sterilized complete culture media (RPMI 1640 media, 10% low endotoxin heat-inactivated FBS, 1 mM Penicillin-Streptomycin). PBMCs were pelleted via centrifugation at 300 x g for 10 min at room temperature. Pellets were gently resuspended in 10 mL of 37°C MACS buffer (PBS, 0.5% bovine serum albumin, 20 mM EDTA, pH 7.2), then viability and cell count were obtained with a hemocytometer using Trypan blue (1:20 dilution) exclusion to ascertain viability.
- Following cryorecovery, CD3+ T cells were isolated from total PBMCs using REAlease® CD3 MicroBead Kit, human (Miltenyi, 130-117-038) following the manufacturer's instructions with slight modifications. PBMCs were centrifuged at 300 x g for 10 min at room temperature, supernatant was aspirated, and pellets were gently resuspended in 40 µL of separation buffer (PBS, 0.5% bovine serum albumin, 2mM EDTA, pH 7.2) per 10 million total cells. 10 µL of REAlease CD3-Biotin were added per 10 million total cells, mixed well, and samples were incubated at room temperature for 5 minutes. 100 µL of REAlease Anti-Biotin Microbeads (CD3, human) were added per 10 million total cells, mixed well, and samples were incubated at room temperature for 5 minutes. Samples were diluted to a total volume of 2 mL with separation buffer then passed through pre-wetted LS columns (Miltenyi, 130-042-401) in a QuadroMACS™ Separator (Miltenyi, 130-091-051). Columns were washed 3 times with 3 mL of separation buffer, and the flow-through was set aside at 4°C for isolation of monocytes. LS columns were removed from the magnetic separator and flushed twice with 5 mL of REAlease Bead Release buffer to release bead-bound

CD3+ cells. CD3+ samples were mixed well and incubated at room temperature for 5 minutes. Then, CD3+ samples were centrifuged at 300 x g for 10 min at 4°C, supernatant was aspirated, pellets were gently resuspended in 5 mL separation buffer. 100 µL of REAlease Release Reagent was added to each sample, mixed well, and then CD3+ cells were counted using a hemocytometer with Trypan blue (1:20 dilution) to ascertain viability.
- Monocytes were isolated from the flow-through of CD3- cells using Pan Monocyte Isolation Kit, human (Miltenyi, 130-096-537) following the manufacturer's instructions with slight modifications. Cells were centrifuged at 300 x g for 10 min at 4°C, supernatant was aspirated, and pellets were gently resuspended in 45 µL of cold separation buffer per 10 million total cells. 15 µL of FcR blocking reagent and 18.75 µL of Biotin-antibody cocktail was added per 10 million total cells, samples were mixed well, and then cells were incubated for 5 minutes at 4°C. 45 µL of cold separation buffer and 30 µL of Anti-Biotin Microbeads were added per 10 million total cells, samples were mixed well, and then cells were incubated for 5 minutes at 4° C. Samples were diluted to a total volume of 2 mL with cold separation buffer then passed through pre-wetted LS columns in a QuadroMACS™ Separator. Columns were washed 3 times with 3 mL of separation buffer, and the flow-through containing purified monocytes was counted on a hemocytometer using Trypan blue (1:20 dilution) to ascertain viability.
- For each study participant, 1 million purified T cells per treatment condition were plated to a 24-well culture plate and allowed to rest for 2 hours at 37°C, 5% CO2, 95% relative humidity. After resting, cells were treated with either vehicle or 3.125 µL Dynabeads™ Human T-Activator CD3/CD28 (Gibco, 11161D) for 72 hours. Likewise, 500,000 purified monocytes per treatment condition were plated to a 24-well culture plate and allowed to rest for 2 hours at 37°C, 5% CO2, 95% relative humidity. After resting, monocytes were treated with either vehicle or 200U human IFNγ (Peprotech, 300-02) for 72 hours.
- After the 72-hour stimulation, cells were harvested and centrifuged at 300 x g for 10 min at 4°C. Supernatant was collected to quantify cytokine secretion (described below). Cell pellets were gently resuspended in 200 µL of cold PBS and transferred to a v-bottom 96-well plate (Sigma, CLS3896-48EA). Samples were centrifuged at 300 x g for 5 min at 4°C.
o 50% of the cells from each condition were processed for the unfixed cell flow cytometry panel, described as follows: Cells were resuspended in 200 µL of complete growth media containing 1 µM MitoTracker™ Red CMXRos (Invitrogen, M7512),1 µM MitoTracker™ Green FM (Invitrogen, M7514), and 1 µM BMV109 Pan Cathepsin Probe (Vergent Biosciences, 40200-200). Cells were incubated for 1 hour at 37°C in the dark. Samples were centrifuged at 300 x g for 5 min at 4°C. Cell pellets were resuspended in PBS and washed x 2 by centrifugation at 300 x g for 5 min at 4°C. Cells were resuspended in 50 µL of Live/Dead Fixable Violet stain (diluted 1:2000 in PBS, Invitrogen, L34962) and incubated in the dark at room temperature for 30 min. Cells were centrifuged at 300 x g for 5 min at 4°C washed in PBS x 2. Cells were resuspended in 50 µL of PBS containing diluted antibodies (see Table 2 for T cell panel, see Table 3 for monocyte panel) and incubated in the dark at 4°C for 20 min. Cells were centrifuged at 300 x g for 5 min at 4°C washed in FACS buffer (PBS, 0.5 mM EDTA, 0.1% sodium azide) x 3 before analysis via flow cytometry on a FACSymphony™ A3 cytometer (BD Biosciences).
o The other 50% of the cells from each condition were processed for the fixed cell flow cytometry panel, described as follows: Cells were resuspended in 200 µL of complete growth media, and LysoTracker™ Red DND-99 (Invitrogen, L7528) was added to reach a final concentration of 500 nM for T cells or 200 nM for monocytes. Cells were incubated for 1 hour at 37°C in the dark. Samples were centrifuged at 300 x g for 5 min at 4°C. Cell pellets were resuspended in PBS and washed x 2 by centrifugation at 300 x g for 5 min at 4°C. Cells were resuspended in 50 µL of Live/Dead Fixable Violet stain (diluted 1:2000 in PBS, Invitrogen, L34962) and incubated in the dark at room temperature for 30 min. Cells were centrifuged at 300 x g for 5 min at 4°C washed in PBS x 2. Cells were resuspended in 50 µL of PBS containing diluted antibodies (see Table 2 for T cell panel, see Table 3 for monocyte panel) and incubated in the dark at 4°C for 20 min. Cells were centrifuged at 300 x g for 5 min at 4°C and washed x 2 in PBS. Cells were re-suspended and fixed in 100 µL of 1% paraformaldehyde (PFA) at 4°C in the dark for 30 min. Cells were washed 2 x with PBS, then resuspended in 100 µL of permeabilization buffer (eBiosciences, 00-8333-56) and incubated on ice for 15 min. Anti-pT73 Rab10 antibody (Abcam, ab241060) was added to each well at 0.55 µg per well and incubated at room temperature and protected from light for 30 min. Cells were centrifuged at 300 x g for 5 min at 4°C washed in PBS x 2. Cells were resuspended in 100 µL of PBS containing 1% normal goat/donkey serum, 2% BSA and 1:1000 AF488 donkey anti-rabbit secondary (Thermo Fisher, A-21206) and incubated at room temperature and protected from light for 30 min. Cells were centrifuged at 300 x g for 5 min at 4°C washed in PBS x 2. Cells were resuspended in 100 µL of PBS containing 1% normal goat/donkey serum, 2% BSA 1:100 anti-LRRK2 AF700 antibody and incubated at 4°C covered for 20 min. Cells were centrifuged at 300 x g for 5 min at 4°C, and then washed in FACS buffer × 3 before analysis via flow cytometry on a FACSymphony™ A3 cytometer (BD Biosciences).

Instrument

BD FACSymphony™ A3 cytometer was used for peripheral immune cell analysis.

Software

The flow cytometry acquisition software used was BD FACSDiva 9.3.1. The analysis software used was FlowJo version 10.10.0

Cell population abundance

The abundance of the relevant cell populations was determined based on gating strategy using fluorescence-minus-one controls (FMOCs) on PBMCs from healthy controls. The gating strategy and marker definitions are described in the research article and Supplementary Information. The relative frequencies of CD4+, CD8+, and CD8-dim T cells and monocyte subtype populations (classical, CD14+CD16-; intermediate, CD14+CD16+; nonclassical, CD14dimCD16+) for each patient group are provided in Figure 4 and Supplementary Figure 5.

Gating strategy

The gating strategy we used is provided in the Supplementary Information and was generated using FMOCs with PBMCs from healthy controls. We first gated to exclude cellular debris, then gated on singlets, and living cells. For T cell analysis, we gated to include the CD3+ population and then gated around CD4+, CD8+, and CD8-dim cells. For monocytes, we gated around classical monocytes (CD14+CD16-), intermediate monocytes (CD14+CD16+), and nonclassical (CD14dimCD16+). After cell type groupings were accomplished, all cell populations were gated around being positive or negatively stained for the various fluorescent antibodies and probes described in the antibodies section of the reporting summary.

☒ Tick this box to confirm that a figure exemplifying the gating strategy is provided in the Supplementary Information.
